# Transcriptional and biochemical responses of monoacylglycerol acyltransferase-mediated oil synthesis and associated senescence-like responses in *Nicotiana benthamiana*

**DOI:** 10.3389/fpls.2014.00204

**Published:** 2014-05-26

**Authors:** Uday K. Divi, Anna El Tahchy, Thomas Vanhercke, James R. Petrie, Jose A. Robles-Martinez, Surinder P. Singh

**Affiliations:** ^1^CSIRO Food Futures National Research FlagshipCanberra, ACT, Australia; ^2^Bioinformatics, CSIRO Plant IndustryCanberra, ACT, Australia

**Keywords:** *Nicotiana benthamiana*, monoacylglycerol, diacylglycerol, triacylglycerol, acyltransferase, oil increase, senescence, differential gene expression

## Abstract

Triacylglycerol (TAG) accumulates in plant seeds as a major renewable source of carbon for food, fuel and industrial feedstock. Approaches to enhance TAG content by altering lipid pathways and genes in vegetative parts have gained significant attention for biofuel and other applications. However, consequences of these modifications are not always studied in detail. In an attempt to increase TAG levels in leaves we previously demonstrated that a novel substrate, monoacylglycerol (MAG), can be used for the biosynthesis of diacylglycerol (DAG) and TAG. Transient expression of the *Mus musculus* monoacylglycerol acyltransferases *MGAT1* and *2* in the model plant *Nicotiana benthamiana* increased TAG levels at 5 days post-infiltration (dpi). Here we show that increased TAG and DAG levels can be achieved as early as 2 dpi. In addition, the *MGAT1* infiltrated areas showed senescence-like symptoms from 3 dpi onwards. To unravel underlying molecular mechanisms, Illumina deep sequencing was carried out (a) for *de-novo* assembling and annotation of *N. benthamiana* leaf transcripts and (b) to characterize *MGAT1-responsive* transcriptome. We found that *MGAT1*-responsive genes are involved in several processes including TAG biosynthesis, photosynthesis, cell-wall, cutin, suberin, wax and mucilage biosynthesis, lipid and hormone metabolism. Comparative analysis with transcript profiles from other senescence studies identified characteristic gene expression changes involved in senescence induction. We confirmed that increased TAG and observed senescence-symptoms are due to the MAG depletion caused by *MGAT1* activity and suggest a mechanism for *MGAT1* induced TAG increase and senescence-like symptoms. The data generated will serve as a valuable resource for oil and senescence related studies and for future *N. benthamiana* transcriptome studies.

## Introduction

Demand for production and use of biofuels is rapidly increasing and current agricultural practices are unable to produce sufficient amounts cost-effectively. Multiple approaches are being employed to develop plants with altered carbon and energy content to meet this demand. Accumulation of energy-dense lipids like triacylglycerols (TAG) in the vegetative parts of plants has attracted significant attention due to the potential to produce large amounts of energy rich biomass (Vanhercke et al., 2014a,b). Approaches to increase TAG levels in diverse plant tissues have targeted different steps of TAG biosynthesis and storage that can be generally divided into (a) “Push”- mobilizing carbon flux to increase fatty acid biosynthesis, (b) “Pull”—TAG assembly, and (c) “Protect”—increasing TAG storage/decreasing TAG breakdown (Vanhercke et al., 2013a,b). Examples of the “Push approach” include ectopic expression of transcription factors (LEC1, LEC2 and WRI1) that control seed development and maturation (Santos Mendoza et al., 2005; Andrianov et al., 2010; Liu et al., 2010) as well as diverting carbon flow from starch to lipid biosynthesis by silencing ADP-glucose-pyrophosphorylase (AGPase) in plant leaves (Sanjaya et al., 2011). “Pull approaches” consist of overexpression of acetyl-CoA carboxylase (Klaus et al., 2004) and overexpression of diacylglycerol acyltransferase (DGAT) and other enzymes involved in TAG assembly (Bouvier-Navé et al., 2000; Jako et al., 2001; Weselake et al., 2008; Petrie et al., 2012). Finally, retaining fatty acid (FA) content (“Protect approach”) by blocking FA movement to peroxisomes or via other mechanisms can also increase TAG levels in leaves (Xu et al., 2005; Slocombe et al., 2009). Many studies which combine several of these approaches (Andrianov et al., 2010; Sanjaya et al., 2011; Vanhercke et al., 2013a) have provided “proof of concept” results for oil increase, although the TAG levels achieved are not yet necessarily of industrial relevance. However, recently substantial progress had been made in this direction by metabolic engineering of plant leaves for high energy dense oil-seed like TAG (Vanhercke et al., 2014a).

In plants diacylglycerol (DAG), the immediate precursor of TAG, is synthesized mainly by: (1) Kennedy pathway for *de novo* DAG synthesis (Kennedy, 1961), and (2) transfer of phosphocholine head group from phosphatidylcholine (PC) to PC-derived DAG by phosphatidylcholine:diacylglycerol cholinephosphotransferase (PDCT) (Lu et al., 2009). Another pathway that involves the conversion of monoacylglycerol (MAG) to DAG by monoacylglycerol acyltransferase (MGAT) has been well characterized in animals (Yen et al., 2002; Cao et al., 2003). Although the presence of MAG in plant tissues has been reported (Perry and Harwood, 1993; Yang et al., 2002), it is only believed to serve as a precursor for cuticular wax biosynthesis (Li-Beisson et al., 2013). Recently, a plant MGAT gene and an OLEOSIN (OLEO3) isolated from peanut were shown to enhance TAG accumulation when over-expressed in *Saccharomyces cerevisiae* (Parthibane et al., 2012; Vijayaraj et al., 2012). In addition, the concept of oil increase in vegetative tissue *via* a MAG pathway was recently demonstrated by (Petrie et al., 2012). The authors showed that heterologous expression of mouse MGATs (*MGAT1* and *2*) in *Nicotiana benthamiana* leaves can lead to utilization of MAG for DAG synthesis, thereby significantly increasing TAG accumulation. TAG levels were increased 9.2-fold by *MGAT1* and 7.3-fold by *MGAT2* at 5 days post-infiltration (dpi) in the infiltrated leaves.

In addition to increased TAG, we found that *MGAT1* infiltrated leaf areas of *N. benthamiana* turn yellow at 3 dpi and exhibit senescence-like symptoms by 5 dpi. These symptoms are specific to *MGAT1* infiltration and can be reduced by MAG supplementation or inhibition of MGAT activity. In order to better understand the molecular mechanisms underlying the observed symptoms and TAG increase, we undertook a global gene expression study of *MGAT1*-mediated responses in the *N. benthamiana* transient leaf infiltration system. This system has been proven particularly useful for the metabolic engineering of lipid biosynthesis pathways (Wood et al., 2009; Petrie et al., 2010; Vanhercke et al., 2013b) and for functional confirmation of novel genes (Dussert et al., 2013). Availability of genetic resources will further broaden the scope for this model plant to understand the consequences of gene/pathway manipulation. Efforts in this direction have been initiated with the draft genome being recently published (Bombarely et al., 2012) and the transcriptome sequenced from a pooled library representing nine different tissues (Nakasugi et al., 2013). Here we describe the lipid pathway related transcripts of *N. benthamiana* leaf tissue and employed RNA-seq to identify genes differentially expressed in *MGAT1* infiltrated leaves. This study provides a comprehensive overview of cellular metabolic processes caused by the utilization of MAG for TAG synthesis and associated senescence-like response resulting from transient expression of mouse *MGAT1* in *N. benthamiana*.

## Results and discussion

### Physiological and biochemical responses of MGAT1 infiltration

**Figure 1:**
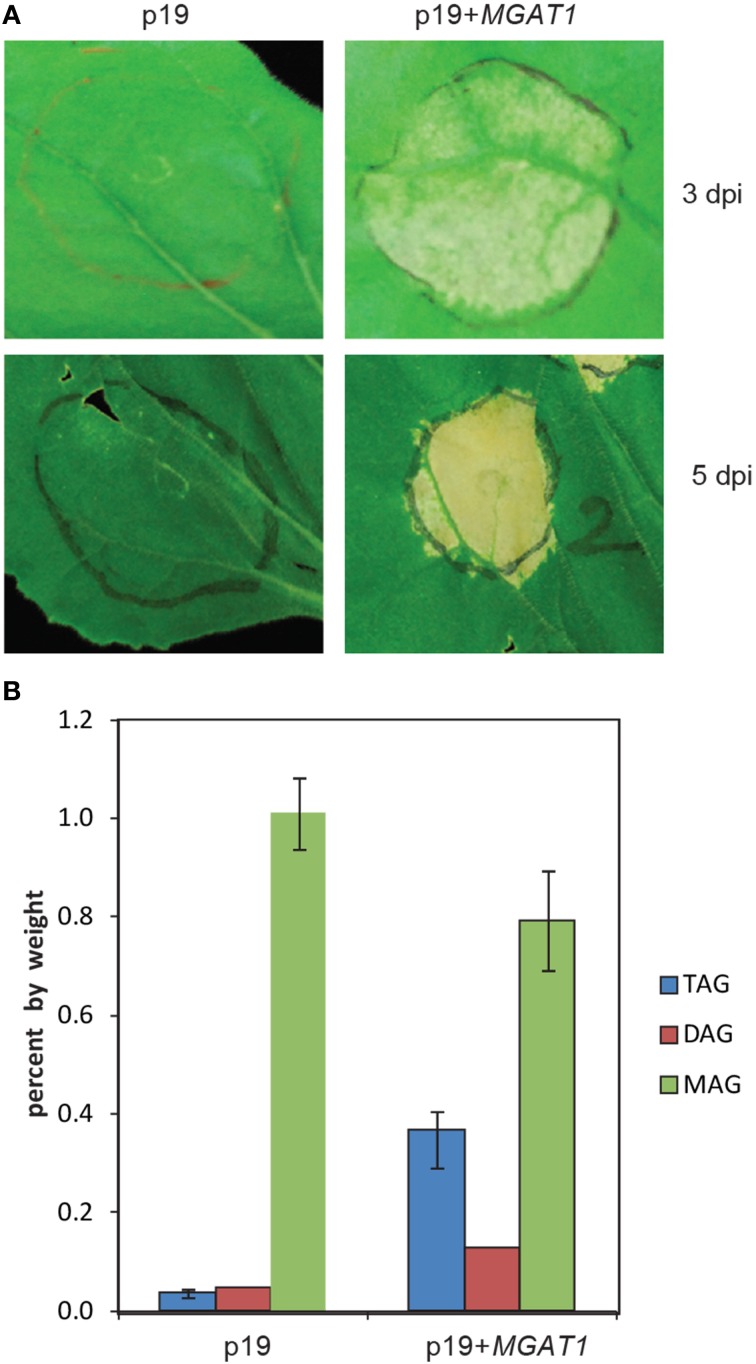
**Physiological and biochemical responses of monoacylglycerol acyltransferase (*MGAT1*) transient expression in *Nicotiana benthamiana* leaves. (A)** Senescence symptoms observed in leaf tissue infiltrated with *MGAT1* at 3 days post-infiltration (dpi) and 5 dpi. Infiltrations were performed along with p19 viral suppressor protein as control (Petrie et al., 2012). **(B)** Lipid analysis showing monoacylglycerol (MAG), diacylglycerol (DAG) and triacylglycerol (TAG) levels in p19 and p19+*MGAT1* infiltrated leaf spots, harvested at 2 dpi. Data shown are average of three replicates and error bars represent standard error.

Transient expression of *M. musculus MGATs (MGAT1* or *MGAT2)* is known to increase TAG accumulation by 5 days post-infiltration (dpi) in *N. benthamiana* leaves (Petrie et al., 2012). However, the *MGAT1*-infiltrated areas showed cell-death by 5 dpi (Figure 1A), with senescence-like symptoms being visible from 3 dpi onwards (Figure 1A). Surprisingly, the *MGAT2* infiltrated leaves show no senescence symptoms even at 5 dpi. Differences in expression patterns, functions and biochemical activities between mammalian MGATs have been reported (Cao et al., 2003, 2007). Further, we have shown previously that MGAT1 displays considerable DGAT side-activity with 58% of the labeled DAG converted to TAG by MGAT1 in contrast with only 6% conversion by MGAT2 (Petrie et al., 2012). Hence, we focused this study on understanding the relationship between transient *MGAT1* expression and the observed senescence-like responses. As a first step, we measured the TAG levels in *MGAT1* infiltrated leaves before the onset of senescence symptoms. Lipid analysis on *MGAT1* infiltrated leaves at an early time point of 2 dpi showed a 4-fold increase in TAG levels (Figure 1B). In addition, DAG levels were also increased while a clear decrease in MAG levels was observed indicating its conversion to DAG. Since TAG accumulation occurs at 2 dpi and senescence symptoms do not start to appear, this early time point was deemed suitable for gene expression studies.

### *De novo* transcriptome assembly and annotation

RNA was extracted from the leaves of 6-week old un-infiltrated plants for *de novo* transcriptome assembly. Illumina sequencing followed by processing of raw reads produced a total of 55 million clean reads (Table 1A). Assembling of the reads using Trinity (Grabherr et al., 2011) produced 92,857 non-redundant contigs out of which 77,679 were singletons and 15,178 were distinct clusters (Table 1A). The length distribution of contigs is shown in Supplementary Figure 1. The mean contig length was 503 bp with a N50 weightage of 706 for the assembly (Table 1A). The contig sequences can be downloaded from CSIRO data access portal (http://dx.doi.org/10.4225/08/524ABB9C42D91).

Table 1**Summary of *de novo* assembling and annotation of *N. benthamiana* contigs**.

**Table:** 

**A**	
**SUMMARY OF *N. BENTHAMIANA* TRANSCRIPTOME SEQUENCES GENERATED BY ILLUMINA HiSeqTM2000**	
Total raw reads	58,558,070
Total clean reads	55,100,052
Total contigs	92,857
Singletons	77,679
Distinct clusters	15,178
Mean length	503
N50	706

**Table:** 

**B**	
**Database**	**No. of *N. benthamiana* contigs annotated**
Non-redundant (nr)	48,952
SwissProt	31,572
KEGG	32,030
COG	13,972
GO	23,568
Total contigs annotated	49,180

*(A) Summary of sequencing and de novo assembling output. (B) Summary of N. benthamiana contigs annotated by different databases*.

The 92,857 contigs were searched against various public databases using BLASTX (1e^−5^). The number of transcripts annotated by each database is summarized in Table 1B. The NCBI non-redundant (nr) database (ftp://ftp.ncbi.nlm.nih.gov/blast/db/) search retrieved annotations for 48,952 contigs. Function annotations for 31,572 contigs were obtained from Swiss-Prot (Magrane and Consortium, 2011) and “Clusters of Orthologous Groups of Proteins” (COG; http://www.ncbi.nlm.nih.gov/COG) database search annotated 13,972 contigs. Gene Ontology (GO) annotation terms were retrieved for 23,568 contigs using Blast2GO (Conesa et al., 2005). Thus, a total of 49,180 contigs were annotated from all the databases indicating that nearly 52% of the transcripts were annotated. The list of contigs and their annotations from each database are described in Supplementary data 1. The COG functional class distribution and categorization into the GO categories are illustrated in Supplementary Figure 2. For metabolic pathway annotations, the contigs were searched against Kyoto Encyclopedia of Genes and Genomes database (KEGG; http://www.genome.jp/kegg/). The enriched pathways and number of transcripts related to each pathway are illustrated in Supplementary Figure 3. About 34.5% (32,030) of transcripts were annotated to the KEGG pathways, the majority of which were under the broad category of metabolic pathway (21.28%). Biosynthesis of secondary metabolites (9.62%), plant hormone signal transduction (6.19%) and plant-pathogen interaction (5.68%) were the other major pathway categories (Supplementary Figure 3). From the pathway analysis, *N. benthamiana* contigs related to lipid pathways are identified and summarized in Table 2.

**Table 2:** **Summary of *N. benthamiana* contigs related to different lipid pathways identified by KEGG pathway analysis**.

**KEGG Pathway**	**Number**	**Percentage**	**Pathway ID**
Fatty acid biosynthesis	81	0.33	ko00061
Fatty acid metabolism	121	0.50	ko00071
Glycerophospholipid metabolism	543	2.24	ko00564
Ether lipid metabolism	345	1.43	ko00565
Sphingolipid metabolism	115	0.48	ko00600
Glycosphingolipid biosynthesis—ganglio series	63	0.26	ko00604
Biosynthesis of unsaturated fatty acids	114	0.47	ko01040

The identified transcripts and their annotations were confirmed by randomly BLASTing a number of contig sequences against the *N. benthamiana* transcriptome database (http://sydney.edu.au/science/molecular_bioscience/sites/benthamiana/). No disagreement in annotation was observed between the matched sequences from both studies.

### Identification of leaf acyl-lipid metabolism genes of *N. benthamiana*

**Figure 2:**
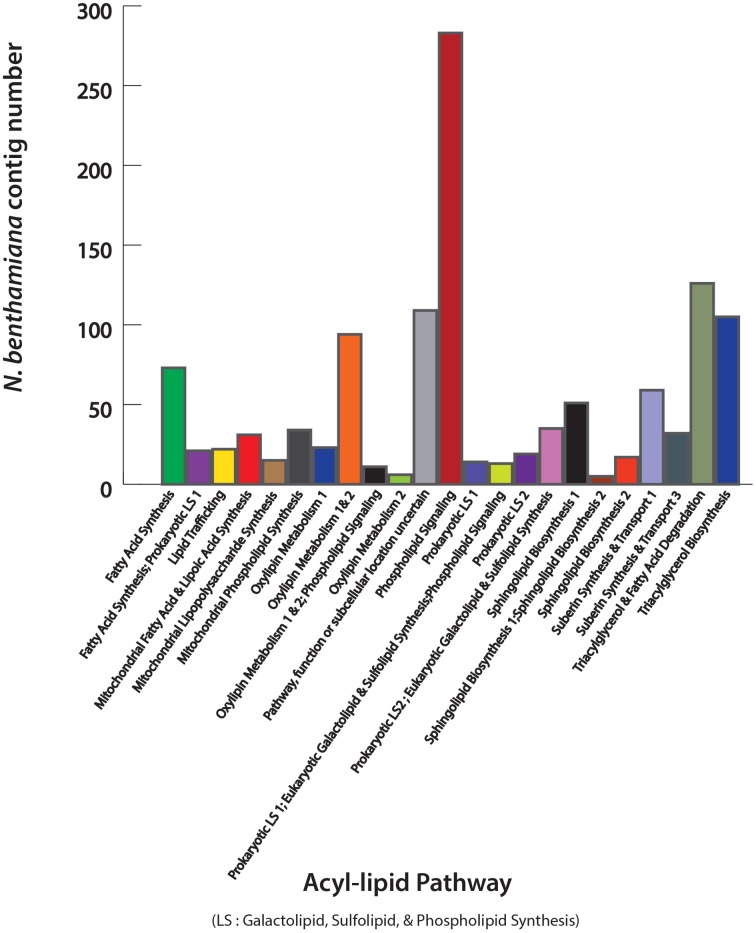
**Identification of leaf acyl-lipid metabolism transcripts of *N. benthamiana***. Contigs mapped to *A. thaliana* sequences were searched against “The Arabidopsis Acyl-Lipid Metabolism” website (ARALIP; http://aralip.plantbiology.msu.edu) to identify acyl-lipid related transcripts. Distribution of identified transcripts into different acyl-lipid metabolism categories and number of contigs in each category is illustrated.

Acyl-lipids include a wide range of lipid classes with diverse functions in cell membranes and sub-cellular organelles (Li-Beisson et al., 2010). Identification of acyl-lipid genes of *N. benthamiana* is important in the context that this model plant can be used extensively for manipulating lipid pathways in leaf tissue. The *N. benthamiana* contig sequences were mapped against the Arabidopsis TAIR10 protein sequences (http://www.arabidopsis.org/) using BLASTX (1e^−5^) in CLC Genomics Workbench 5 (http://www.clcbio.com). Identified contigs with an *Arabidopsis thaliana* homolog were analyzed against acyl-lipid metabolism gene list obtained from “The Arabidopsis Acyl-Lipid Metabolism” website (ARALIP; http://aralip.plantbiology.msu.edu). A total of 1648 acyl-lipid contigs of *N. benthamiana* leaf were identified. The distribution of these transcripts into 23 different lipid-related function categories and transcript count for each category are shown in Figure 2. In plants, the majority of the seed oil is stored as TAG. The *N. benthamiana* transcripts that are involved in TAG biosynthesis were identified and are described in Supplementary Table 1. A list of all the contigs identified with a role in acyl-lipid metabolism is shown in Supplementary data 2.

### Transcriptional responses of MGAT1 infiltration

In order to understand the molecular mechanisms underlying *MGAT1*-mediated TAG accumulation and senescence-like responses, RNA-seq analysis was performed on *MGAT1* infiltrated vs. control infiltrated leaves. The early time point of 2 dpi showing TAG accumulation but not senescence symptoms along with an additional time point of 1 dpi was selected for identifying differentially expressed genes. RNA was extracted from the control (p19) and *MGAT1*-infiltrated leaf areas, pooled from at least 10 different leaves for each treatment at each time point, and Illumina sequencing reads were obtained. The expression levels were estimated as Reads Per Kilobase of Exon per Million mapped reads (RPKM) by mapping against the *de novo* assembled transcriptome (92,857 contigs) in CLC Genomics. Differentially expressed transcripts were identified according to the RNA-seq workflow in CLC Genomics and also by using a R package DESeq (Anders and Huber, 2010). No significant differences were observed in the fold changes derived from both tools. Gene lists derived from DESeq containing *p*-values (cut-off *p* < 0.05) adjusted for multiple testing by Benjamini-Hochberg procedure were used for further analysis. After applying a fold change (FC) cut-off of ≥2/≤−2 (*p* < 0.05), 178 genes were up-regulated and 134 genes were down-regulated at 1 dpi. At 2 dpi, 846 genes were up-regulated and 2434 genes down-regulated (Supplementary Figure 4A). *A. thaliana* homologs were identified for 152 (1 dpi) and 482 (2 dpi) up-regulated genes, and 103 (1 dpi) and 1890 (2 dpi) down-regulated genes. It is note-worthy that, similar to *MGAT1* response at 2 dpi, several senescence studies noted a larger number of genes being down-regulated than up-regulated genes under senescing conditions (Troncoso-Ponce et al., 2013). A scatter plot using the log2 FC values of 4 genes estimated by RNA-seq and qRT-PCR is shown in Supplementary Figure 4B. Further, the expression of *MGAT1* in infiltrated leaves was confirmed by RT-PCR (Supplementary Figure 4C).

### Gene ontology (GO) and pathway (MapMan) annotations of differentially expressed genes

**Figure 3:**
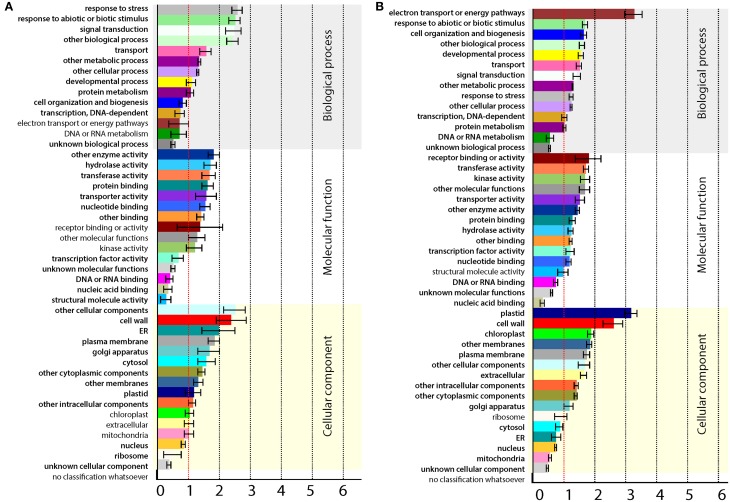
**Gene ontology (GO) annotations of differentially expressed genes (DEG)**. Classification SuperViewer tool at The Bio-Analytic Resource (BAR) for Plant Biology (http://bar.utoronto.ca) was used. **(A,B)** GO term annotation of up-regulated **(A)** and down-regulated genes **(B)**. Significantly enriched terms are highlighted in bold.

Further analysis was performed on contigs having an *A. thaliana* homolog by taking advantage of the available resources. Since major changes in gene expression were observed at 2 dpi only, this category of genes was selected for further analysis. After removing duplications in annotations, 1666 transcripts were found to be changed at 2 dpi, out of which 1333 were down-regulated and 333 were up-regulated. The up- and down-regulated genes of 2 dpi were analyzed for enriched GO terms (*p* < 0.05) using Classification SuperViewer tool at The Bio-Analytic Resource (BAR) for Plant Biology (http://bar.utoronto.ca), as illustrated in Figure 3. The up-regulated genes were highly enriched in stress response, abiotic or biotic stimulus and signal transduction biological responses (Figure 3A). This indicates that, in addition to accumulation of increased TAG, *MGAT1* infiltrated areas were also experiencing and responding to stress. A significant number of down-regulated genes were located in plastid, chloroplast and cell-wall (Figure 3B), suggesting that cellular processes localized to these organelles could be negatively impacted by *MGAT1*. Indeed the electron transport and energy pathways were highly enriched in down-regulated genes indicating a suppressive effect on these processes by *MGAT1*.

### Metabolism overview

**Figure 4:**
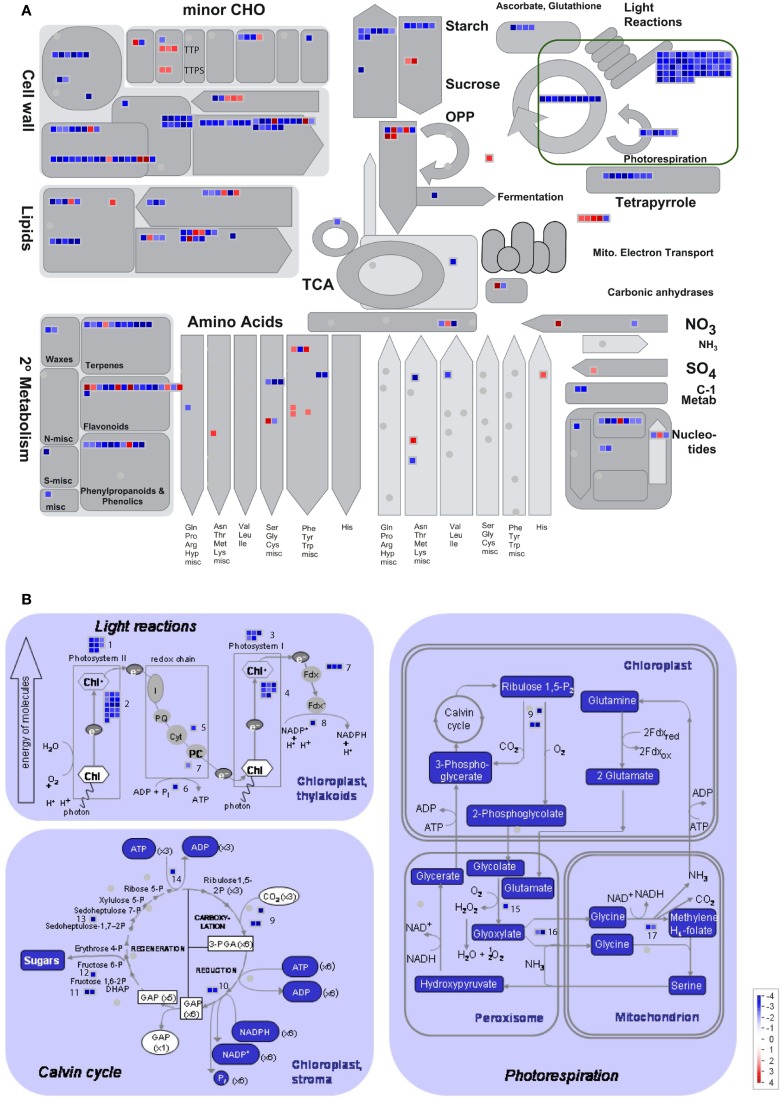
**MapMan pathway overview analysis of DEG. (A)** Overview of *MGAT1* effect on transcripts related to different pathways generated by “Metabolism overview” analysis in MapMan. Fold changes (Log2) are indicated by the colour scale. Distribution of transcripts into different pathways and their expression trend in each pathway is illustrated. **(B)** Effect of *MGAT1* on photosynthesis (PS) related transcripts. All PS related genes were down-regulated. The number of transcripts, their distribution into sub-classes of PS and expression trend are illustrated. The 17 sub-classes and the genes in each sub-class are described in Supplementary Table 2.

To provide an overview of *MGAT1* effect on individual metabolic pathways, significantly changed genes (≥2/≤−2-fold; *p* < 0.05) along with their fold changes were mapped on pathway bins of MapMan 3.5.1R2 (http://mapman.gabipd.org). Metabolism overview revealed that the majority of *MGAT1* responsive genes map to cell-wall, lipids and PS bins (Figure 4A). Most of the cell-wall related genes including cellulose synthases (*CESA 2–6*), cellulose synthase like (*CSL)* genes, and arabinogalactan proteins (AGPs) such as salt overly sensitive 5 (*SOS 5*) involved in cell-wall biosynthesis (Xu et al., 2008) were down-regulated. Cell-wall modifying genes like expansins, xyloglucan endotransglycosylase *(XETs)* and pectin esterases were also down-regulated. Down-regulation of cell-wall synthesis and modification genes indicates a possible disruption in cell-wall integrity. Genes involved in carbohydrate (CHO) metabolism were also affected by *MGAT1* (Figure 4A). While many major CHO genes related to sucrose and starch metabolism were down-regulated, a cell-wall invertase (*AtcwINV2*; At3g52600) and a vacuolar invertase (At1g62660) were up-regulated. Decline of photosynthesis (PS) initiates senescence and a role for invertases in this process has been demonstrated (Dickinson et al., 1991; Ding et al., 1993). Minor CHO related genes including trehalose-6-phosphate phosphatases (*TPPs*) and two trehalose-phosphate synthases (*TPS 5* and *11*) were also up-regulated by *MGAT1* (Figure 4A). An overview of *MGAT1* effect on PS is illustrated in Figure 4B and corresponding genes are summarized in Supplementary Table 2. In agreement with the GO analysis showing down-regulated terms of electron transport, energy and plastid, PS showed largest down-regulation effect by *MGAT1*, as compared to other pathways. The collapse of PS, cell-wall, major CHO metabolism and electron transport/ATP synthesis are major indicators of senescence induction that can cause the visible symptoms observed at 3 dpi.

### Genes involved in MGAT1 induced tag biosynthesis

**Figure 5:**
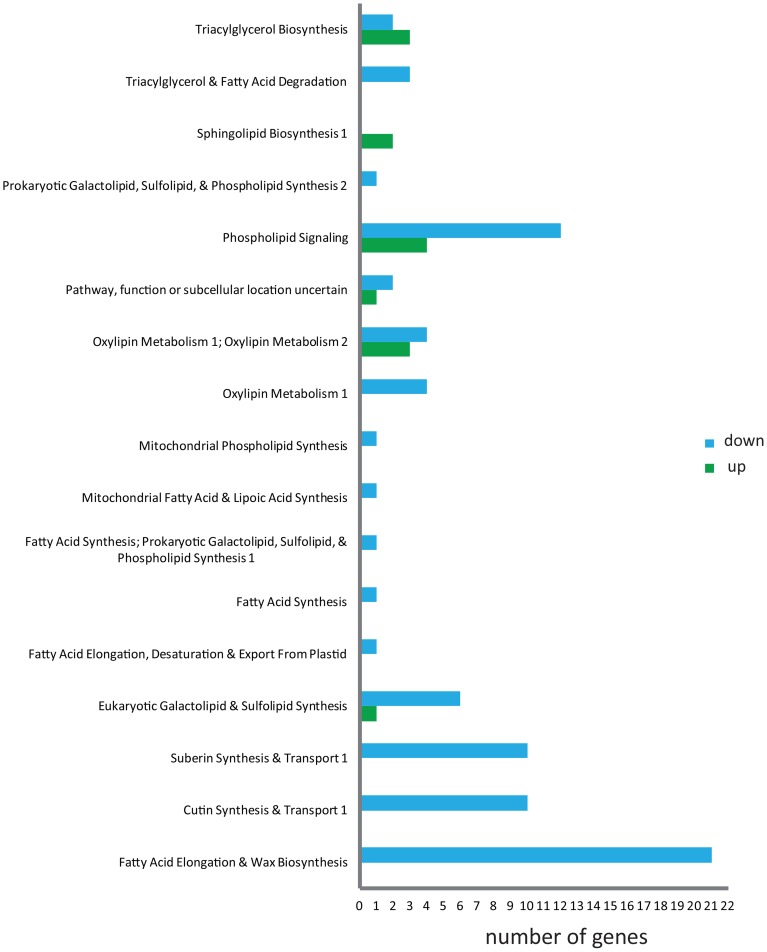
**Identification of *MGAT1* DEGs involved in acyl-lipid metabolism**. Distribution of genes differentially expressed by *MGAT1* into different categories of acyl-lipid metabolism is illustrated. Down-regulation (blue) of transcripts in majority of classes including FA elongation, cutin, suberin and wax biosynthesis can be seen, while TAG biosynthesis contains more up-regulated (green) genes.

The *N. benthamiana* acyl-lipid transcripts identified in the study were analyzed for differentially regulated genes and lipid processes induced by *MGAT1* infiltration. Expression of 94 transcripts mapped to ARALIP genes were altered by *MGAT1* expression, of which only 12 were up-regulated (Supplementary Table 3). The only lipid process enriched with up-regulated genes was TAG biosynthesis, while genes involved in FA synthesis and modification, TAG degradation, FA elongation, wax biosynthesis, cutin and suberin synthesis and transport were all found to be down-regulated (Figure 5). These responses are indicative of the induction of TAG biosynthesis machinery by *MGAT1* expression while trancripts involved in FA degradation, synthesis and transport of wax, suberin and cuticular structures appear to be suppressed. Two TAG biosynthesis related genes, diacylglycerol acyltransferase (*DGAT1)* and phosphatidylcholine:diacylglycerol cholinephosphotransferase (*PDCT*) were up-regulated. FA degradation genes down-regulated included two MAG lipases (*MAGL*) and a TAG lipase *(TAGL)*. PDCT and the transiently expressed *MGAT1* are involved in synthesizing DAG from PC and MAG, respectively. DAG is the only substrate of DGAT1 for conversion to TAG. Transient expression of *MGAT1* and the subsequent up-regulation of *PDCT* and *DGAT1* could explain the increased DAG and TAG levels detected in *MGAT1* infiltrated areas in combination with depletion in MAG levels compared to the negative control (p19). Down-regulation of MAG and TAG lipases could result in reduced neutral lipid turnover thereby contributing to a net increase in TAG levels.

### Expression profiles of MGAT1 responsive lipid-related genes during senescence

**Figure 6:**
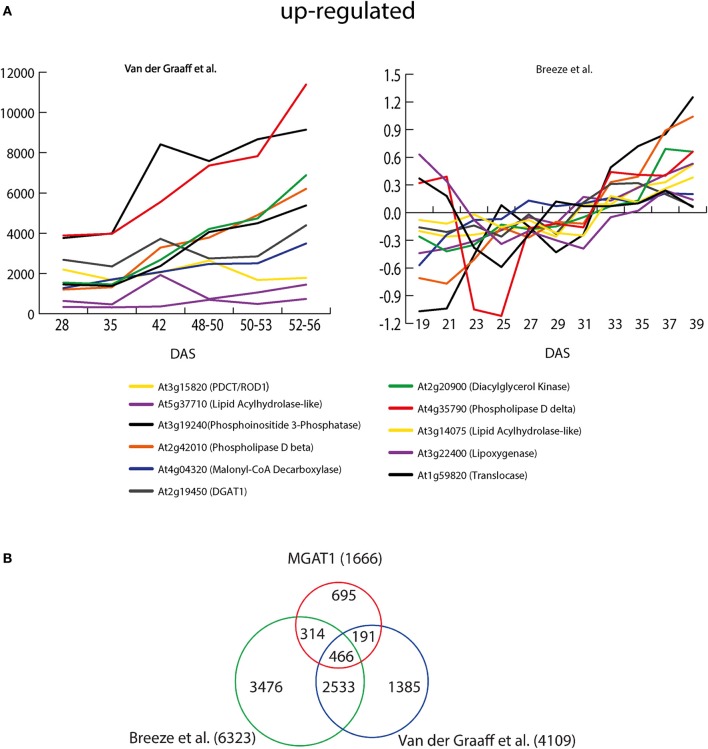
***MGAT1* and senescence responsive transcripts. (A)** Expression profiles of *MGAT1* responsive lipid related genes during senescence. Senescence profiles from two earlier studies (Van Der Graaff et al., 2006; Breeze et al., 2011) were downloaded from Troncoso-Ponce et al. (2013) database and shown. Eleven out of twelve lipid-related genes up-regulated by *MGAT1* were also up-regulated during progression of senescence shown as days after sowing (DAS). **(B)** Comparative analysis of *MGAT1* and senescence responsive transcriptome. Venn diagram showing gene overlap between *MGAT1*, Breeze et al. and Vander Graff et al.

Recently, Troncoso-Ponce et al. (2013) have developed a database by compiling and plotting the senescence responsive profiles of lipid-related genes from three independent studies (Schmid et al., 2005; Van Der Graaff et al., 2006; Breeze et al., 2011). We chose Breeze et al. (2011) and Van Der Graaff et al. (2006) studies only to compare the *MGAT1* responsive profiles of the lipid-related genes as Schmid et al. study was not specifically directed at senescence. Breeze et al., analyzed expression pattern of *A. thaliana* genes from 19 days after sowing (DAS) to 39 DAS. Van der Graaff et al. studied expression profiles of developmental leaf senescence (NS), darkening-induced senescence in intact leaves (DIS) and senescence in dark-incubated detached leaves (DET) at different stages. Out of the 12 lipid-related genes identified to be up-regulated by *MGAT1*, 11 genes were also up-regulated during senescence in both studies (Figure 6A). A large number (69) of lipid-related genes were down-regulated by *MGAT1*. While no clear trend emerged, the majority of these genes were also down-regulated in both Breeze et al. (78%) and Van der Graaff et al. (52%) senescence studies (Supplementaty Figure 5). These results indicate that transcriptional changes caused by *MGAT1* infiltration are similar to those induced by senescence as discussed below.

One of the characteristic responses of lipid-related genes during senescence is a co-ordinated decrease in the transcripts for FA synthesis and desaturation, while the glycerolipid assembly transcripts including *DGAT1* increase in expression (Troncoso-Ponce et al., 2013). In line with this, transcripts involved in FA synthesis such as *KASI*, *KASII*, β-ketoacyl-CoA synthase (*KCS)* and long-chain acyl-CoA synthase (*LACS2*), as well as *FAD3* and *5* involved in FA desaturation were down-regulated by *MGAT1*, while *DGAT1* and *PDCT* involved in glycerolipid assembly were up-regulated. In contrast to the up-regulation of some glycerol-3-phosphate acyltransferases (*GPAT9* and *GPAT1*) during senescence (Troncoso-Ponce et al., 2013), *GPAT4* required for MAG accumulation was down-regulated by *MGAT1*. This finding indicates that in addition to utilizing MAG for conversion to DAG, *MGAT1* also affects MAG synthesis by decreasing *GPAT4* expression which can result in further reduction of MAG levels in *MGAT1* infiltrated leaves. Up-regulation of genes encoding various lipases except for phospholipase D (PLD) and genes involved in β-oxidation pathway is a feature of lipid turnover during natural senescence (Thompson et al., 1998; Troncoso-Ponce et al., 2013). Somewhat unexpectedly, transcripts of two putative MAG lipases and a TAG lipase were down-regulated by *MGAT1* expression, while no change was observed for β-oxidation genes. In addition, PLD-β and PLD-δ transcripts were up-regulated by *MGAT1*. Several putative ABC transporters involved in FA transport were also down-regulated by *MGAT1*. Blocking of FA transport to peroxisomes by mutating an ABC transporter resulted in increased TAG levels during senescence (Slocombe et al., 2009). Thus, while several *MGAT1* induced lipid-responses are similar to senescence responses including increase in DGAT1 transcripts and higher TAG levels, it appears that *MGAT1* also blocks FA turnover resulting in reduced TAG turnover.

### Comparative analysis of MGAT1 and senescence responsive gene expression

To understand the relation between *MGAT1* expression and senescence, the entire set of *MGAT1*-responsive genes was compared with senescence response gene lists from both Breeze et al. (6323 genes) and Van der Graaff et al. (4109 genes) studies. The expression of 466 out of 1666 (27.9%) *MGAT1*-responsive genes were also changed in both studies. While 314 genes showed a specific overlap with Breeze et al., 191 *MGAT1*-responsive genes overlapped specifically with Van der Graaff et al. As expected, a larger proportion (2532) of genes was common between the two senescence studies. About 47.1% (695) of *MGAT1*-responsive genes were specific to *MGAT1* and were unaffected in either study (Figure 6B). Interestingly, this subset includes transcription factors *MYB5* and *TRANSPARENT TESTA GLABRA (TTG2)*, found to be down-regulated by *MGAT1*. In *A. thaliana*, MYB5 and *TTG2* are key components in regulation of mucilage biosynthesis (Western et al., 2004). While *myb5-1* mutants exhibit loss of mucilage and enhanced oil accumulation (Shi et al., 2012), *TTG2* is required for the expression of GLABRA2 (GL2), a negative regulator of seed oil accumulation (Shen et al., 2006; Shi et al., 2012). Taken together, it is apparent from these results that in addition to a good overlap with senescence related genes, *MGAT1*-specific changes were also abundant.

In order to perform comparisons based on expression pattern and associated functions, cluster analysis was performed on gene lists from all three studies using MapMan. Genes from each study were categorized into up- and down-regulated and given a value of “2” and “−2” respectively. Genes that did not change in a given condition or gene list were given a value of “1.” Using eucledian distance metric, 15 clusters were generated (Supplementary Figure 6). Interestingly, 216 genes were down-regulated in all studies and grouped to Cluster A1. Out of these 24% belong to PS. Genes related to cell-wall, lipid and protein metabolism, secondary metabolism and RNA regulation were also present in this cluster. Clusters A2–5 consists of genes down-regulated by *MGAT1* and in one or more other studies. Clusters A3 (56 genes) and A4 (30 genes) together represent the second largest set of genes. This set includes many transcription factors and signaling kinases along with FA synthesis genes *KASII* and *LACS* (A3). These profiles point to the similarities of *MGAT1* responses to natural senescence in affecting PS and lipid pathways. Cluster B1 genes were up-regulated in almost every condition. This subset includes *SRG3*, *PR4 (PATHOGENESIS-RELATED 4)*, heat shock protein (*HSP17.8-CI*), *HSP70*, *SIP4* (*SOS3-INTERACTING PROTEIN 4*) and *CML37* (*CALMODULIN LIKE 37*) with known roles in biotic and abiotic stresses. The key gene for ethylene biosynthesis, *ACS2* (At1g01480) is also present in this cluster. Up-regulation of these genes in all conditions is indicative of stress and senescence related responses and increased synthesis of the senescence hormone ethylene. Genes up-regulated by *MGAT1* but either down-regulated or unchanged in one or more of the other conditions were grouped in clusters B2 to B5. This set includes a senescence marker gene *YLS9* (*YELLOW-LEAF-SPECIFIC GENE 9*) that is responsive to late senescence, dark, ethylene and ABA (Yoshida et al., 2001), *PLDBETA1* (*PHOSPHOLIPASE D BETA 1*) [cluster B3]; the ABA biosynthesis gene, *NCED3* (*NINE-CIS-EPOXYCAROTENOID DIOXYGENASE 3*), two Ethylene signaling genes *EBF2* (*EIN3-BINDING F BOX PROTEIN 2*) and *RRTF1 (REDOX RESPONSIVE TRANSCRIPTION FACTOR 1*), and the transcription factor STZ (salt tolerance to zinc finger) [Cluster B4]. STZ is a key factor controlling the expression of senescence related NAC transcription factors (Breeze et al., 2011). Cluster C1 consists of 5 genes specifically up-regulated by *MGAT1* which include a cell-wall synthesis related gene MATERNAL EFFECT EMBRYO ARREST (MEE31) and a peroxidise (PER12). Genes down-regulated specifically by *MGAT1* were grouped in clusters C2 (31 genes) and C3 (30 genes). Among the C3 genes, 7 were transcription factors which include two ethylene signaling genes *ETHYLENE-RESPONSIVE ELEMENT BINDING PROTEIN* (*ATEB*, At3g16770) and *ETHYLENE-INSENSITIVE 3* (*EIN3*). Genes down-regulated by *MGAT1* and in DET were grouped in cluster C4. Important in this cluster was an ABA biosynthesis gene, *NCED4* that was down-regulated in NS as well. These results point to senescence-like gene expression changes in response to *MGAT1* infiltration, the majority of which affect PS. Similarities in phytohormone-related gene expression patterns were also apparent.

### MGAT1 and leaf senescence

**Figure 7:**
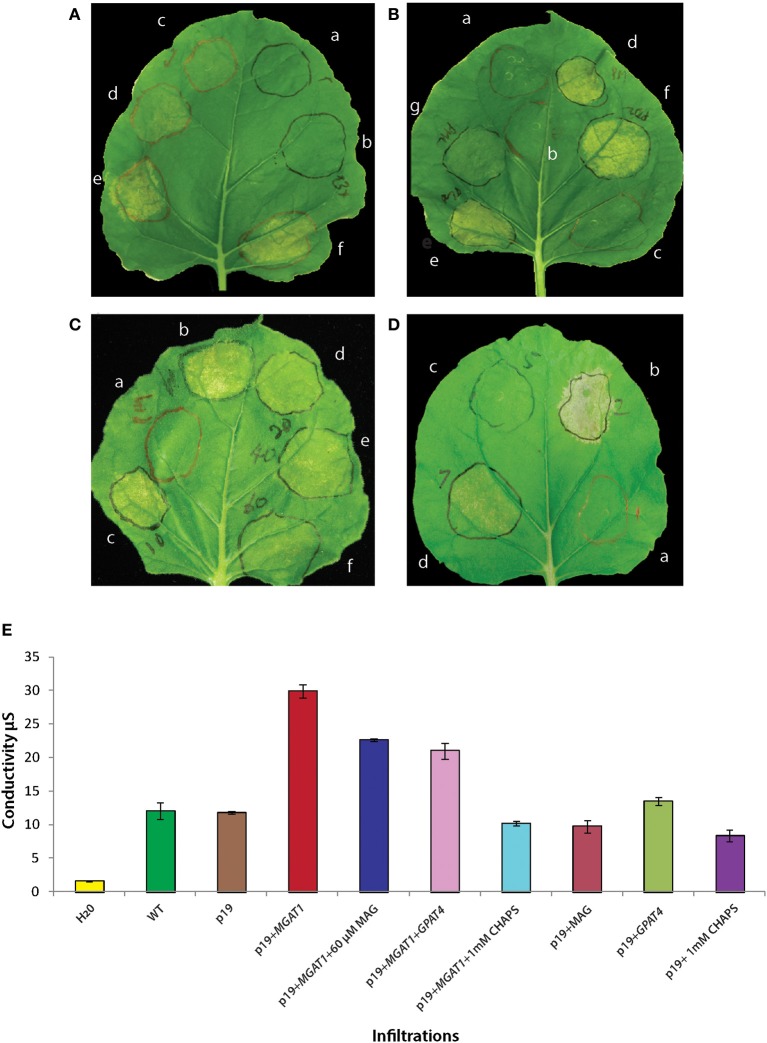
**Effect of *MGAT1* expression and MAG depletion on observed senescence symptoms. (A)** Leaf spots infiltrated with two different concentrations of p19 [OD 0.125 **(Aa)** and OD 0.375 **(Ab)**] and four different concentrations of p19 + *MGAT1* [0.03 OD **(Ac)**, 0.0625 OD **(Ad)**, 0.125 OD **(Ae)** and 0.25 OD **(Af)**]. The severity of visible symptoms is correlated to *MGAT1* concentration. **(B)** Co-infiltration of CHAPS or diacylglycerol acyltransferase (*DGAT1*) with *MGAT1*. No senescence symptoms are detected in the control treatments—p19 **(a)**, p19 + 1 mM CHAPS **(b)** and p19 + *DGAT1* **(c)**. Senescence induced by *MGAT1* **(d)** is abolished by including 1 mM CHAPS in infiltration mix containing *MGAT1* **(g)**. No effect was observed when *DGAT1* was co-infiltrated [0.125 OD **(e)** and 0.25 OD **(f)**]. **(C)** MAG supplementation: 2-Oleoylglycerol was added to the infiltration buffer to obtain 10 μM **(c)**, 20 μM **(d)**, 40 μM **(e)**, or 60 μM **(f)** final concentrations. Senescence was considerably reduced in the presence of 60 μM MAG **(f)** in comparison to *MGAT1* alone **(b)**. Increase in MAG concentration was accompanied by a decrease in visible senescence **(c–f)**. **(D)** Co-infiltration of a glycerol-3-phosphate acyltransferase (*AtGPAT4*) with *MGAT1* **(d)** considerably reduced visible senescence as compared to the *MGAT1* control **(b)**. Infiltration of *AtGPAT4* by itself **(c)** did not induce senescence. **(E)** Ion-conductivity measurement of *MGAT1* induced senescence. Ion conductivity of infiltrated leaf discs was measured by floating in water. Water with no leaf discs floated (H_2_0) and un-infiltrated (WT) leaf discs served as controls. Data shown are average of three replicates and error bars represent standard error.

To confirm the role of *MGAT1* in the observed senescence, *N. benthamiana* leaves were infiltrated with four different concentrations of *Agrobacterium tumefaciens* (AGL1) containing *MGAT1*. A clear correlation between *MGAT1* concentration and leaf yellowing was observed at 3 dpi (Figures 7Ac–f). No significant yellowing was visible at 0.03 OD concentration (Figure 7Ac), while yellowing appeared at 0.0625 OD (Figure 7Ad) and was much more pronounced at 0.125 (Figure 7Ae) and 0.25 OD (Figure 7Af). Infiltrations with *A. tumefaciens* cultures containing p19 at OD 0.125 and 0.375 did not result in a senescence phenotypic response (Figures 7Aa,b). Although the above results indicated a clear role of *MGAT1* in the induction of senescence-like responses, we further confirmed this hypothesis by blocking the MGAT1 activity. The zwitterionic detergent, CHAPS has been used extensively to inhibit the MGAT and DGAT activities of MGAT enzymes (Cao et al., 2007; Vijayaraj et al., 2012). Co-infiltration of 1 mM CHAPS along with *MGAT1* did not show senescence symptoms in the infiltrated area (Figures 7Bg) while symptoms were observed in the *MGAT1* infiltrated areas of the same leaves (Figure 7Bd). These results clearly show that the activity of infiltrated *MGAT1* is required for observed senescence-like symptoms.

### Role of MAG in MGAT1-induced senescence-like response

Although we confirmed the role of *MGAT1* in the induction of senescence-like symptoms, the involvement of MAG depletion or DAG/TAG accumulation was still not clear. Since accumulation of DAG or TAG has not always been associated with senescence, we hypothesized that decrease in MAG levels by MGAT1 activity could be involved. However, since it is known that co-infiltration of *DGAT1* together with *MGAT2* leads to the efficient conversion of accumulated DAG to TAG by the co-expressed *DGAT1* (Petrie et al., 2012), we first tested the effect of *DGAT1* co-infiltration with *MGAT1* on observed senescence. As shown in Figure 7B, no significant changes in visible senescence were observed between *MGAT1* (Figure 7Bd) infiltration and co-infiltration with *DGAT1* at two different concentrations of 0.125 and 0.25 OD (Figures 7Be–f). *DGAT1* itself did not cause any senescence (Figure 7Bc). These results, although not conclusive, indicate that DGAT1 activity had no effect on senescence. The fact that the observed senescence was comparable between *MGAT1* and *MGAT1*+*DGAT1* pointed to a relation between reduced MAG levels and senescence. Indeed, supplementation with an 18:1 sn-2 MAG (2-Oleoylglycerol) at different concentrations (10–60 μM) during infiltration alongside *MGAT1* rescued the observed senescence symptoms in a concentration dependent manner (Figures 7Cc–f) as compared to *MGAT1* only (Figure 7Cb). To further confirm the role of MAG depletion in senescence, *GPAT4* gene encoding a sn-2 specific acyltransferase involved in MAG synthesis was co-infiltrated with *MGAT1*. Similar to MAG supplementation, *GPAT4* co-infiltration (Figure 7Dd) reduced the severity of senescence symptoms induced by *MGAT1* (Figure 7Db). A tight correlation between observed senescence and supplementation by both exogenous and genetic modes are indicative of a role for MAG depletion in *MGAT1* induced senescence. It is noteworthy that *GPAT4* was also down-regulated by *MGAT1*.

### Ion-conductivity measurement of MGAT1 induced senescence-like response

In order to quantify the cell damage, electrical conductivity of infiltrated leaf areas was measured as described in methods. Leaves were infiltrated with *MGAT1*, *MGAT1* + 60 μM MAG, *MGAT1* + *GPAT4* and *MGAT1* + 1 mM CHAPS alongside appropriate controls for each treatment. Leaf discs harvested from infiltrated leaves were floated overnight in sterile water for measuring conductivity. Similar to the visual observations, *MGAT1* infiltrated leaves showed highest conductivity (30 μS). Both MAG (60 μM) supplementation and *GPAT4* co-infiltration reduced the conductivity by 24.5% and 29.7% respectively, whereas a 34% reduction was observed in presence of 1 mM CHAPS (Figure 7E). All controls including p19 showed a mean conductivity of 15 μS. These results show that MAG depletion due to conversion to DAG by *MGAT1* can induce senescence-like symptoms that can be rescued by providing additional MAG substrate. Measurement of TAG, DAG and MAG levels under different senescence rescue treatments should provide further biochemical evidence for the role of MAG in senescence.

## Conclusions

**Figure 8:**
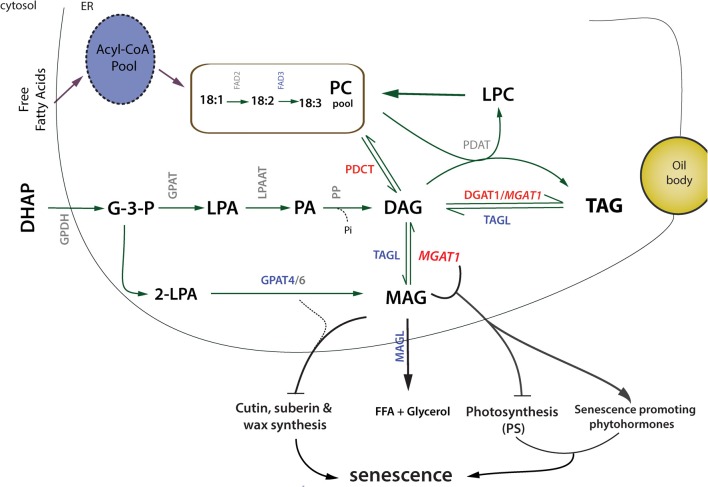
**Schematic overview of *MGAT1*-induced TAG increase and senescence-like response**. Up- (red) and down-regulated (bue) genes are highlighted. Transient expression of *MGAT1* and subsequent up-regulation of *PDCT* possibly result in enhanced DAG synthesis from MAG and PC, respectively. DAG is subsequently converted to TAG due to the co-ordinated up-regulation of *DGAT1* and DGAT side-activity of *MGAT1*. Down-regulation of TAG and MAG lipases (*TAGL* and *MAGL*) indicate decreased FA degradation and increased TAG retention. Down-regulation of cutin, wax and suberin biosynthesis transcripts including *GPAT4* and diversion of MAG for TAG synthesis is likely to affect biosynthesis of surface lipids. In addition, down-regulation of photosynthesis genes and up-regulation of senescence hormone related genes indicate a co-ordinated mechanism that might initiate senescence-like response.

We have shown that recruiting MAG for TAG synthesis via *MGAT1* transient expression results in increased TAG accumulation as early as 2 dpi and also induces senescence-like response in *N. benthamiana* infiltrated leaves. A *de novo* transcriptome data set was generated from leaf tissue to study the underlying molecular changes. This data set allowed identification of genes involved in lipid pathways of the model plant *N. benthamiana* and can serve as a resource for future transcriptomic studies in this system. Using this transcriptome as reference we have identified genes differentially expressed by *MGAT1* infiltration. Similarities in transcriptional responses to *MGAT1* expression and development of senescence were identified by comparative analysis. These similarities include a majority of down-regulated genes, down-regulation of entire set of photosynthesis related genes, activation of senescence hormone responses, decrease in FA synthesis and desaturation related transcripts. On the other hand, genes involved in glycerolipid assembly such as *PDCT* and *DGAT1* were found to be up-regulated. These results provide a mechanism for MGAT-mediated TAG synthesis in which transient expression of *MGAT1* and subsequent up-regulation of *PDCT* results in enhanced DAG synthesis from MAG and PC, respectively. Subsequently, DAG is efficiently converted to TAG due to (a) the co-ordinated up-regulation of *DGAT1* and (b) the DGAT side-activity of MGAT1 (Figure 8). In addition, interesting *MGAT1* responses were identified including the down-regulation of transcription factors *MYB5* and *TTG2* involved in the regulation of mucilage biosynthesis. The senescence related responses of *MGAT1* include dramatic decrease in PS-related genes, down-regulation of cutin, suberin and wax biosynthesis genes including *GPAT4* (Figure 8). Further, we demonstrate that *MGAT1* inhibition, addition of MAG or co-infiltration of *GPAT4* can reduce the observed senescence as indicated by a decrease in ion-conductivity. Taken together, these findings suggest that MAG conversion to DAG by *MGAT1* leads to MAG depletion affecting diverse biological processes via gene expression changes thereby initiating a senescence-like response. DAG has been suggested as a signaling molecule (Dong et al., 2012). While its role in *MGAT1* mediated senescence-like response cannot be ruled out, the *N. benthamiana* system will allow further characterization of the role of different DAG molecular species under senescence-promoting and -rescue conditions. Finally, *MGAT1* transient expression could provide a valuable tool for the study of surface lipids biosynthesis such as cutin, wax and suberin as well as plant-pathogen research due to the ability to induce controlled cell-death in the leaves of a model plant.

## Materials and methods

### Transient expression in *N. benthamiana*

All infiltrations of *Agrobacterium tumefaciens* (AGL1) for transient gene expression in *N. benthamiana* were performed on 6 week old leaves as described previously (Voinnet et al., 2003; Wood et al., 2009). RNA extracted from 6 weeks old un-infiltrated *N. benthamiana* leaves was used for *de novo* transcriptome assembly. For differential gene expression analysis, *MGAT1* and p19 genes were transiently expressed. Infiltrated areas from at least 10 different leaves were pooled after 1 and 2 dpi separately and frozen for RNA extraction. 10 mM CHAPS (3-[(3-Cholamidopropyl)dimethylammonio]-1-propanesulfonate); (Sigma-Aldrich, USA) solution prepared in deionized water was included in the infiltration buffer to yield a final concentration of 1 mM. For MAG supplementation, 2-Oleoylglycerol (Sigma-Aldrich) dissolved in acetonitrile was added to the infiltration buffer to obtain 10, 20, 40, or 60 μM final concentrations. The mouse *MGAT1* and *A. thaliana DGAT1* and *GPAT4* sequences used in this study were described previously (Yang et al., 2002; Petrie et al., 2012).

### Transcriptome analysis

#### Illumina sequencing

RNA (20 μg) extracted from frozen leaf tissues using RNeasy Plant Mini kit (Qiagen, Hilden, Germany) was used for cDNA library preparation and sequencing at Beijing Genome Institute (BGI, Shenzhen, China). Briefly, poly (A) mRNA was isolated with Oligo(dT) attached beads and subjected to fragmentation. First strand cDNA synthesis by random hexamers was followed by second-strand synthesis and purification (QiaQuick PCR purification kit, Qiagen), end repair and adapter ligation. Equimolar concentrations of single end, 100 bp libraries generated after size selection and PCR enrichment were sequenced in a single lane of Illumina HiSeq™ 2000 system.

#### De novo assembly and annotation

The RNA-Seq reads generated were initially processed to remove the adapter sequences, ambiguous reads (5%) and low quality sequences (Q20). Reads were *de novo* assembled to contigs using Trinity (Grabherr et al., 2011). Singletons and clusters with isoforms were identified by building de Brujin graphs using Chrysalis module in Trinity. The contigs were mapped against public databases including nr (ftp://ftp.ncbi.nlm.nih.gov/blast/db/), Swiss-prot (http://www.uniprot.org/), COG (http://www.ncbi.nlm.nih.gov/COG) and KEGG (http://www.genome.jp/kegg/) using BLASTX (*E*-value cutoff 1e^−5^) for functional annotation. Gene Ontology (GO) annotation terms were obtained by using Blast2GO (Conesa et al., 2005). The contigs were aligned against the *A. thaliana* protein database TAIR 10 (http://www.arabidopsis.org/) by downloading the sequences and BLASTing (cut-off 1e^−5^) in CLC Genomics Workbench 5 (http://www.clcbio.com). The *A. thaliana* acyl-lipid gene list was obtained from “The Arabidopsis Acyl-Lipid Metabolism” website (ARALIP; http://aralip.plantbiology.msu.edu). The GO term enrichment analysis was performed by Classification SuperViewer tool at BAR (http://bar.utoronto.ca).

#### Differential gene expression analysis

RNA-Seq reads from the *MGAT1* and p19 (control) RNA samples were loaded on to CLCBio Genomics workbench 5 for expression analysis. The expression values of transcripts in each sample were measured as RPKM (Mortazavi et al., 2008) in the RNA-seq workflow of CLC Genomics. The parameters used include maximum number of 2 mismatches and minimum read count of 10. Fold change analyses was performed in CLC Genomics by setting up a two group comparison experiment. Differential expression analysis was also carried using DESeq package (1.14.0) in R (2.15.3). The nbinomTest function was used to estimate fold changes which employs Benjamini-Hochberg procedure for false discovery rate (FDR) control and outputs the fold changes and the *p*-values for statistical significance (Anders and Huber, 2010). Transcripts with 2-fold up- or down-regulation (*p* ≤ 0.05) and having assigned *A. thaliana* genes were further analyzed. The *A. thaliana* gene expression data during natural senescence (Troncoso-Ponce et al., 2013) was used for comparing the *MGAT1* responsive expression profiles at day 2. The *MGAT1* responsive gene list (1666 genes), Supplementary data set of Breeze et al. and Supplement Table 3 of Vander Graaff et al. were used for comparative analysis and for cluster analysis in MapMan 3.5.1R2 (http://mapman.gabipd.org).

### qRT-PCR analysis

Total RNA (1 μg) was converted to cDNA using First-Strand cDNA synthesis mix (OriGene Inc., Australia). Diluted cDNA (0.2X) was used for quantitative real-time PCR by Bio-Rad CFX Real-Time System (BIO-RAD, *USA*) using iQSYBR Green Supermix (BIO-RAD). Primer validation was performed for all target genes with five dilutions of template concentration along with the *N. benthamiana ACTIN* and *GAPDH* control genes. Reactions were carried out with initial denaturation at 95°C for 3 min, followed by 35 cycles of 95°C for 10 s, 58°C for 30 s and 68°C for 30 s. Relative fold differences were determined by ΔΔ C_T_ method (Livak and Schmittgen, 2001). Primer sequences and description of the target and control sequences are provided in Supplementary Table 4.

### Electrolyte leakage

Six week old *N. benthamiana* leaves were infiltrated as described above. Infiltrated leaf discs (0.5 cm diameter) were harvested after 2 days, rinsed twice (2–3 min) with deionized water and subsequently floated on 15 mL of deionized water with shaking (80 rpm) at room temperature. The electrolyte leakage in the solution was measured after 17 h of floating using a conductivity meter (CD-4301, Lutron). All measurements were done on three biological replicates by pooling samples from six plants (two plants per replicate). Values are the average of three replicates and error bars represent standard error.

### Lipid analysis

Leaf discs (2 cm diameter) were cut from 6 different plants (two plants per replicate). Total lipids were extracted from freeze dried tissue as previously reported (Petrie et al., 2012), run on a TLC plate (20 cm^2^, Silica gel 60, Merck) and developed using a two-step solvent system. Samples were first run to 12 cm in chloroform:methanol:acetic acid:water (68:22:6:4, v:v:v:v), followed by a second separation in hexane:diethyl ether:acetic acid (70:30:1, v:v:v). TAG, DAG and MAG bands were visualized using iodine vapor and isolated from the silica gel. Fatty acid methyl esters were prepared from each isolated lipid fraction and analyzed by GC-FID (7890A GC, Agilent Technologies, Palo Alto, CA, USA) equipped with a 30 m BPX70 column (SGE, Austin, TX, USA) as described previously (Petrie et al., 2012). Peaks were integrated with Agilent Technologies ChemStation software (Revion B.04.03).

## Author contributions

Uday K. Divi conceived of the study, carried out transcriptomic analysis, transient expression studies, conducted data analysis and interpretation and drafted the manuscript. Anna El Tahchy carried out lipid analysis and participated in drafting of the manuscript. Thomas Vanhercke and James R. Petrie participated in design and coordination of the study, data interpretation and critical review of the manuscript. Jose A. Robles-Martinez provided R scripts for differential expression analysis. Surinder P. Singh participated in conceptualization, design and coordination of the study and critical review of the manuscript.

### Conflict of interest statement

The authors declare that the research was conducted in the absence of any commercial or financial relationships that could be construed as a potential conflict of interest.
