# Supplementary material for: Transcriptional and biochemical responses of monoacylglycerol acyltransferase-mediated oil synthesis and associated senescence-like responses in Nicotiana benthamiana
Source: Front Plant Sci. 2014 May 26;5:204. doi: 10.3389/fpls.2014.00204 (PMC4033622; doi:10.3389/fpls.2014.00204)
Supplement: Supplementary file 1 [file DataSheet1.DOCX]

***Supplementary Material***

**Transcriptional and biochemical responses of monoacylglycerol acyltransferase-mediated oil synthesis and associated senescence-like responses in *Nicotiana benthamiana***

**Uday K Divi ^1^*, Anna El Tahchy^1^, Thomas Vanhercke^1^, James R Petrie^1^, Jose A Robles-Martinez^2,3^ and Surinder P Singh^1^.**

^1^CSIRO Food Futures National Research Flagship, Canberra, ACT, Australia.

^2^CSIRO Plant Industry, Bioinformatics, Canberra, ACT, Australia.

^3^Australian Curriculum Assessment and Reporting Authority, Sydney, NSW, Australia.

*** Correspondence:** Uday Divi, CSIRO Plant Industry, PO Box 1600, Canberra, ACT 2601, Australia

[uday.divi@csiro.au](mailto:surinder.singh@csiro.au)

**1. Supplementary Data**

Supplementary Data 1.

Supplementary Data 2.

1. **Supplementary Figures and Tables**

## Suplementary Tables

**Supplementary Table 1.** *N. benthamiana* contigs identified with a role in acyl- lipid metabolism. Contig ID and Corresponding *A. thaliana* gene ID is shown along with Protein family name and EC number.

| ***N.benthamiana* Contig ID** | **Arabidopsis Gene Locus** | **Protein Family Name** | **Abbreviation** | **Isoform/Gene Speciﬁc Abbreviation** | **EC number** |
| --- | --- | --- | --- | --- | --- |
| Unigene20624_Benth | AT3G57650 | 1-Acylglycerol-3-Phosphate Acyltransferase | LPAAT | LPAAT2 | 2.3.1.51 |
| Unigene21787_Benth | AT1G75020 | 1-Acylglycerol-3-Phosphate Acyltransferase | LPAAT | LPAAT4 | 2.3.1.51 |
| Unigene52111_Benth | AT3G18850 | 1-Acylglycerol-3-Phosphate Acyltransferase | LPAAT | LPAAT5 | 2.3.1.51 |
| Unigene60681_Benth | AT3G57650 | 1-Acylglycerol-3-Phosphate Acyltransferase | LPAAT | LPAAT2 | 2.3.1.51 |
| Unigene67881_Benth | AT3G57650 | 1-Acylglycerol-3-Phosphate Acyltransferase | LPAAT | LPAAT2 | 2.3.1.51 |
| Unigene74413_Benth | AT3G18850 | 1-Acylglycerol-3-Phosphate Acyltransferase | LPAAT | LPAAT5 | 2.3.1.51 |
| CL15338.Contig1_Benth | AT3G18850 | 1-Acylglycerol-3-Phosphate Acyltransferase | LPAAT | LPAAT5 | 2.3.1.51 |
| Unigene33412_Benth | AT1G12640 | 1-Acylglycerol-3-Phosphocholine Acyltransferase | LPCAT | LPLAT1/LPCAT1 | 2.3.1.23 |
| Unigene65628_Benth | AT1G12640 | 1-Acylglycerol-3-Phosphocholine Acyltransferase | LPCAT | LPLAT1/LPCAT1 | 2.3.1.23 |
| CL15795.Contig1_Benth | AT1G12640 | 1-Acylglycerol-3-Phosphocholine Acyltransferase | LPCAT | LPLAT1/LPCAT1 | 2.3.1.23 |
| CL2391.Contig1_Benth | AT1G12640 | 1-Acylglycerol-3-Phosphocholine Acyltransferase | LPCAT | LPLAT1/LPCAT1 | 2.3.1.23 |
| Unigene1532_Benth | AT2G30470 | a member of a novel family of B3 domain proteins | Regulatory protein | HSI2/VAL1 |  |
| Unigene2335_Benth | AT2G30470 | a member of a novel family of B3 domain proteins | Regulatory protein | HSI2/VAL1 |  |
| Unigene12152_Benth | AT4G32010 | a member of a novel family of B3 domain proteins | Regulatory protein | HSL1/VAL2 |  |
| Unigene15172_Benth | AT2G30470 | a member of a novel family of B3 domain proteins | Regulatory protein | HSI2/VAL1 |  |
| Unigene16147_Benth | AT2G30470 | a member of a novel family of B3 domain proteins | Regulatory protein | HSI2/VAL1 |  |
| Unigene32016_Benth | AT2G30470 | a member of a novel family of B3 domain proteins | Regulatory protein | HSI2/VAL1 |  |
| Unigene49449_Benth | AT4G32010 | a member of a novel family of B3 domain proteins | Regulatory protein | HSL1/VAL2 |  |
| Unigene55441_Benth | AT4G32010 | a member of a novel family of B3 domain proteins | Regulatory protein | HSL1/VAL2 |  |
| Unigene57666_Benth | AT4G32010 | a member of a novel family of B3 domain proteins | Regulatory protein | HSL1/VAL2 |  |
| Unigene71368_Benth | AT2G30470 | a member of a novel family of B3 domain proteins | Regulatory protein | HSI2/VAL1 |  |
| Unigene77014_Benth | AT2G30470 | a member of a novel family of B3 domain proteins | Regulatory protein | HSI2/VAL1 |  |
| CL14349.Contig1_Benth | AT4G32010 | a member of a novel family of B3 domain proteins | Regulatory protein | HSL1/VAL2 |  |
| CL1766.Contig1_Benth | AT4G32010 | a member of a novel family of B3 domain proteins | Regulatory protein | HSL1/VAL2 |  |
| Unigene881_Benth | AT2G25170 | a SWI/SWF nuclear-localized chromatin remodeling factor of the CHD3 group | Regulatory protein | PKL |  |
| Unigene3684_Benth | AT2G25170 | a SWI/SWF nuclear-localized chromatin remodeling factor of the CHD3 group | Regulatory protein | PKL |  |
| Unigene3685_Benth | AT2G25170 | a SWI/SWF nuclear-localized chromatin remodeling factor of the CHD3 group | Regulatory protein | PKL |  |
| Unigene3686_Benth | AT2G25170 | a SWI/SWF nuclear-localized chromatin remodeling factor of the CHD3 group | Regulatory protein | PKL |  |
| Unigene14360_Benth | AT2G25170 | a SWI/SWF nuclear-localized chromatin remodeling factor of the CHD3 group | Regulatory protein | PKL |  |
| Unigene36079_Benth | AT2G25170 | a SWI/SWF nuclear-localized chromatin remodeling factor of the CHD3 group | Regulatory protein | PKL |  |
| Unigene39583_Benth | AT2G25170 | a SWI/SWF nuclear-localized chromatin remodeling factor of the CHD3 group | Regulatory protein | PKL |  |
| Unigene40758_Benth | AT2G25170 | a SWI/SWF nuclear-localized chromatin remodeling factor of the CHD3 group | Regulatory protein | PKL |  |
| Unigene41205_Benth | AT2G25170 | a SWI/SWF nuclear-localized chromatin remodeling factor of the CHD3 group | Regulatory protein | PKL |  |
| Unigene54716_Benth | AT2G25170 | a SWI/SWF nuclear-localized chromatin remodeling factor of the CHD3 group | Regulatory protein | PKL |  |
| CL443.Contig1_Benth | AT2G25170 | a SWI/SWF nuclear-localized chromatin remodeling factor of the CHD3 group | Regulatory protein | PKL |  |
| CL6339.Contig1_Benth | AT2G25170 | a SWI/SWF nuclear-localized chromatin remodeling factor of the CHD3 group | Regulatory protein | PKL |  |
| CL1648.Contig1_Benth | AT2G25170 | a SWI/SWF nuclear-localized chromatin remodeling factor of the CHD3 group | Regulatory protein | PKL |  |
| Unigene16409_Benth | AT3G51520 | Acyl-CoA : Diacylglycerol Acyltransferase | DGAT | DGAT2 | 2.3.1.20 |
| Unigene47495_Benth | AT2G19450 | Acyl-CoA : Diacylglycerol Acyltransferase | DGAT | DGAT1 | 2.3.1.20 |
| Unigene48502_Benth | AT2G19450 | Acyl-CoA : Diacylglycerol Acyltransferase | DGAT | DGAT1 | 2.3.1.20 |
| Unigene61363_Benth | AT2G19450 | Acyl-CoA : Diacylglycerol Acyltransferase | DGAT | DGAT1 | 2.3.1.20 |
| Unigene61364_Benth | AT2G19450 | Acyl-CoA : Diacylglycerol Acyltransferase | DGAT | DGAT1 | 2.3.1.20 |
| CL1857.Contig1_Benth | AT1G48300 | Acyl-CoA : Diacylglycerol Acyltransferase | DGAT | DGAT3 | 2.3.1.20 |
| CL1305.Contig1_Benth | AT2G19450 | Acyl-CoA : Diacylglycerol Acyltransferase | DGAT | DGAT1 | 2.3.1.20 |
| CL1305.Contig2_Benth | AT2G19450 | Acyl-CoA : Diacylglycerol Acyltransferase | DGAT | DGAT1 | 2.3.1.20 |
| CL12651.Contig1_Benth | AT3G51520 | Acyl-CoA : Diacylglycerol Acyltransferase | DGAT | DGAT2 | 2.3.1.20 |
| CL14620.Contig1_Benth | AT3G51520 | Acyl-CoA : Diacylglycerol Acyltransferase | DGAT | DGAT2 | 2.3.1.20 |
| Unigene4544_Benth | AT3G54320 | AP2/EREBP Transcription Factors ( or is it AP2/ERWEBP) | Regulatory protein | WRI1 |  |
| CL1077.Contig1_Benth | AT3G54320 | AP2/EREBP Transcription Factors ( or is it AP2/ERWEBP) | Regulatory protein | WRI1 |  |
| Unigene34257_Benth | AT1G70670 | Caleosin | CALO |  |  |
| Unigene39324_Benth | AT1G70670 | Caleosin | CALO |  |  |
| Unigene14399_Benth | AT1G74320 | Choline Kinase | CK |  | 2.7.1.32 |
| Unigene20661_Benth | AT1G71697 | Choline Kinase | CK | CK1 | 2.7.1.32 |
| Unigene34999_Benth | AT1G74320 | Choline Kinase | CK |  | 2.7.1.32 |
| Unigene66548_Benth | AT1G71697 | Choline Kinase | CK | CK1 | 2.7.1.32 |
| CL14627.Contig1_Benth | AT1G71697 | Choline Kinase | CK | CK1 | 2.7.1.32 |
| CL15161.Contig1_Benth | AT1G74320 | Choline Kinase | CK |  | 2.7.1.32 |
| CL10377.Contig1_Benth | AT1G71697 | Choline Kinase | CK | CK1 | 2.7.1.32 |
| Unigene9319_Benth | AT2G32260 | Choline-Phosphate Cytidylyltransferase | CCT | CCT1 | 2.7.7.15 |
| Unigene9320_Benth | AT2G32260 | Choline-Phosphate Cytidylyltransferase | CCT | CCT1 | 2.7.7.15 |
| Unigene9321_Benth | AT2G32260 | Choline-Phosphate Cytidylyltransferase | CCT | CCT1 | 2.7.7.15 |
| CL3814.Contig1_Benth | AT2G32260 | Choline-Phosphate Cytidylyltransferase | CCT | CCT1 | 2.7.7.15 |
| CL11989.Contig1_Benth | AT5G60620 | Glycerol-3-Phosphate Acyltransferase | GPAT | GPAT9 | 2.3.1.15 |
| Unigene49238_Benth | AT3G24650 | Homologous to the maize transcription factor Viviparous-1 | Regulatory protein | ABI3 |  |
| CL1749.Contig1_Benth | AT2G29980 | Linoleate Desaturase | FAD3 | FAD3 | 1.14.99.* |
| Unigene16409_Benth | AT3G51520 | Monoacylglycerol Acyltransferase | MAGAT |  | 2.3.1.22 |
| CL12651.Contig1_Benth | AT3G51520 | Monoacylglycerol Acyltransferase | MAGAT |  | 2.3.1.22 |
| CL14620.Contig1_Benth | AT3G51520 | Monoacylglycerol Acyltransferase | MAGAT |  | 2.3.1.22 |
| Unigene11940_Benth | AT2G41540 | NAD-dependent Glycerol-3-Phosphate Dehydrogenase | GPDH | GPDHc1 | 1.1.1.8 |
| Unigene22061_Benth | AT2G41540 | NAD-dependent Glycerol-3-Phosphate Dehydrogenase | GPDH | GPDHc1 | 1.1.1.8 |
| Unigene22062_Benth | AT3G07690 | NAD-dependent Glycerol-3-Phosphate Dehydrogenase | GPDH |  | 1.1.1.8 |
| CL15452.Contig1_Benth | AT2G41540 | NAD-dependent Glycerol-3-Phosphate Dehydrogenase | GPDH | GPDHc1 | 1.1.1.8 |
| CL11062.Contig1_Benth | AT3G07690 | NAD-dependent Glycerol-3-Phosphate Dehydrogenase | GPDH |  | 1.1.1.8 |
| Unigene44798_Benth | AT3G18570 | Oil-Body Oleosin | OBO |  |  |
| Unigene59491_Benth | AT3G27660 | Oil-Body Oleosin | OBO |  |  |
| Unigene9988_Benth | AT3G12120 | Oleate Desaturase | FAD2 | FAD2 | 1.14.99.* |
| Unigene23150_Benth | AT3G12120 | Oleate Desaturase | FAD2 | FAD2 | 1.14.99.* |
| Unigene54449_Benth | AT3G12120 | Oleate Desaturase | FAD2 | FAD2 | 1.14.99.* |
| Unigene75314_Benth | AT3G12120 | Oleate Desaturase | FAD2 | FAD2 | 1.14.99.* |
| Unigene11965_Benth | AT3G09560 | Phosphatidate Phosphatase | PP |  | 3.1.3.4 |
| Unigene50175_Benth | AT3G09560 | Phosphatidate Phosphatase | PP |  | 3.1.3.4 |
| Unigene66636_Benth | AT5G42870 | Phosphatidate Phosphatase | PP |  | 3.1.3.4 |
| Unigene75592_Benth | AT5G42870 | Phosphatidate Phosphatase | PP |  | 3.1.3.4 |
| CL15745.Contig1_Benth | AT3G09560 | Phosphatidate Phosphatase | PP |  | 3.1.3.4 |
| CL6148.Contig1_Benth | AT5G42870 | Phosphatidate Phosphatase | PP |  | 3.1.3.4 |
| CL4998.Contig1_Benth | AT5G42870 | Phosphatidate Phosphatase | PP |  | 3.1.3.4 |
| CL10874.Contig1_Benth | AT3G09560 | Phosphatidate Phosphatase | PP |  | 3.1.3.4 |
| CL5508.Contig1_Benth | AT3G09560 | Phosphatidate Phosphatase | PP |  | 3.1.3.4 |
| Unigene36175_Benth | AT3G15820 | Phosphatidylcholine:diacylglycerol cholinephosphotransferase | PDCT | PDCT/ROD1 | 2.7.8.* |
| Unigene36176_Benth | AT3G15820 | Phosphatidylcholine:diacylglycerol cholinephosphotransferase | PDCT | PDCT/ROD1 | 2.7.8.* |
| Unigene61503_Benth | AT3G15820 | Phosphatidylcholine:diacylglycerol cholinephosphotransferase | PDCT | PDCT/ROD1 | 2.7.8.* |
| CL2795.Contig1_Benth | AT3G15820 | Phosphatidylcholine:diacylglycerol cholinephosphotransferase | PDCT | PDCT/ROD1 | 2.7.8.* |
| Unigene5655_Benth | AT3G44830 | Phospholipid : Diacylglycerol Acyltransferase | PDAT |  | 2.3.1.43 |
| Unigene8030_Benth | AT3G44830 | Phospholipid : Diacylglycerol Acyltransferase | PDAT |  | 2.3.1.43 |
| Unigene27932_Benth | AT5G13640 | Phospholipid : Diacylglycerol Acyltransferase | PDAT | PDAT1 | 2.3.1.43 |
| Unigene43086_Benth | AT3G44830 | Phospholipid : Diacylglycerol Acyltransferase | PDAT |  | 2.3.1.43 |
| Unigene43109_Benth | AT5G13640 | Phospholipid : Diacylglycerol Acyltransferase | PDAT | PDAT1 | 2.3.1.43 |
| Unigene44736_Benth | AT5G13640 | Phospholipid : Diacylglycerol Acyltransferase | PDAT | PDAT1 | 2.3.1.43 |
| Unigene44737_Benth | AT5G13640 | Phospholipid : Diacylglycerol Acyltransferase | PDAT | PDAT1 | 2.3.1.43 |
| Unigene49368_Benth | AT3G44830 | Phospholipid : Diacylglycerol Acyltransferase | PDAT |  | 2.3.1.43 |
| Unigene55299_Benth | AT3G44830 | Phospholipid : Diacylglycerol Acyltransferase | PDAT |  | 2.3.1.43 |
| Unigene63413_Benth | AT5G13640 | Phospholipid : Diacylglycerol Acyltransferase | PDAT | PDAT1 | 2.3.1.43 |
| Unigene69617_Benth | AT5G13640 | Phospholipid : Diacylglycerol Acyltransferase | PDAT | PDAT1 | 2.3.1.43 |
| Unigene73262_Benth | AT5G13640 | Phospholipid : Diacylglycerol Acyltransferase | PDAT | PDAT1 | 2.3.1.43 |
| CL3166.Contig1_Benth | AT5G13640 | Phospholipid : Diacylglycerol Acyltransferase | PDAT | PDAT1 | 2.3.1.43 |

**Supplementary Table 2.** List of photosynthesis related genes differentially expressed by *MGAT1* from MapMan analysis as shown in Figure 4B. Functional description and Log2 fold change (FC) values are also shown.

| **Figure 4B ID** | **BinCode** | **AGI** | **Description** | **MGAT1-Day2 Log2 FC** |
| --- | --- | --- | --- | --- |
| 1 | 1.1.1.1 | At1g15820 | Symbols: LHCB6, CP24 \| LHCB6 (LIGHT HARVESTING COMPLEX PSII SUBUNIT 6); chlorophyll binding \| chr1:5446462-5447859 REVERSE | -3.188 |
|  | 1.1.1.1 | At1g29930 | Symbols: CAB1, AB140, CAB140, LHCB1.3 \| CAB1 (CHLOROPHYLL A/B BINDING PROTEIN 1); chlorophyll binding \| chr1:10478005-10479048 FORWARD | -2.951 |
|  | 1.1.1.1 | At2g05100 | Symbols: LHCB2.1, LHCB2 \| LHCB2.1; chlorophyll binding \| chr2:1823240-1824425 REVERSE | -2.441 |
|  | 1.1.1.1 | At2g34420 | Symbols: LHB1B2, LHCB1.5 \| LHB1B2; chlorophyll binding \| chr2:14522524-14523568 REVERSE | -3.626 |
|  | 1.1.1.1 | At3g27690 | Symbols: LHCB2.4, LHCB2.3, LHCB2 \| LHCB2.3; chlorophyll binding \| chr3:10255947-10257011 FORWARD | -3.202 |
|  | 1.1.1.1 | At4g10340 | Symbols: LHCB5 \| LHCB5 (LIGHT HARVESTING COMPLEX OF PHOTOSYSTEM II 5); chlorophyll binding \| chr4:6408016-6409677 FORWARD | -2.511 |
|  | 1.1.1.1 | At5g01530 | chlorophyll A-B binding protein CP29 (LHCB4) \| chr5:208937-210445 FORWARD | -3.176 |
|  | 1.1.1.1 | At5g54270 | Symbols: LHCB3, LHCB3*1 \| LHCB3 (LIGHT-HARVESTING CHLOROPHYLL B-BINDING PROTEIN 3); structural molecule \| chr5:22038273-22039568 FORWARD | -3.259 |
| 2 | 1.1.1.2 | At1g03600 | photosystem II family protein \| chr1:898876-899655 FORWARD | -2.585 |
|  | 1.1.1.2 | At1g05385 | photosystem II 11 kDa protein-related \| chr1:1582671-1583718 REVERSE | -3.033 |
|  | 1.1.1.2 | At1g06680 | Symbols: PSBP-1, OEE2, PSII-P, OE23 \| PSBP-1 (PHOTOSYSTEM II SUBUNIT P-1); poly(U) binding \| chr1:2047825-2049418 FORWARD | -2.729 |
|  | 1.1.1.2 | At1g44575 | Symbols: NPQ4, PSBS \| NPQ4 (NONPHOTOCHEMICAL QUENCHING); chlorophyll binding / xanthophyll binding \| chr1:16871696-16873383 FORWARD | -2.378 |
|  | 1.1.1.2 | At1g51400 | photosystem II 5 kD protein \| chr1:19052023-19052582 REVERSE | -3.488 |
|  | 1.1.1.2 | At2g06520 | Symbols: PSBX \| PSBX (photosystem II subunit X) \| chr2:2587729-2588358 REVERSE | -3.227 |
|  | 1.1.1.2 | At2g28605 | Encodes a PsbP domain-OEC23 like protein localized in thylakoid (peripheral-lumenal side). \| chr2:12254888-12255837 FORWARD | -3.047 |
|  | 1.1.1.2 | At2g30570 | Symbols: PSBW \| PSBW (PHOTOSYSTEM II REACTION CENTER W) \| chr2:13019028-13020194 REVERSE | -3.909 |
|  | 1.1.1.2 | At2g39470 | Symbols: PPL2 \| PPL2 (PsbP-like protein 2); calcium ion binding \| chr2:16476216-16477843 FORWARD | -3.941 |
|  | 1.1.1.2 | At3g01440 | oxygen evolving enhancer 3 (PsbQ) family protein \| chr3:168435-169618 FORWARD | -3.07 |
|  | 1.1.1.2 | At3g50820 | Symbols: PSBO2, PSBO-2, OEC33 \| PSBO2 (PHOTOSYSTEM II SUBUNIT O-2); oxygen evolving/ poly(U) binding \| chr3:18890876-18892426 REVERSE | -2.758 |
|  | 1.1.1.2 | At3g55330 | Symbols: PPL1 \| PPL1 (PsbP-like protein 1); calcium ion binding \| chr3:20513825-20515351 REVERSE | -2.758 |
|  | 1.1.1.2 | At4g05180 | Symbols: PSBQ, PSBQ-2, PSII-Q \| PSBQ-2; calcium ion binding \| chr4:2671822-2673243 REVERSE | -2.86 |
|  | 1.1.1.2 | At4g28660 | Symbols: PSB28 \| PSB28 (PHOTOSYSTEM II REACTION CENTER PSB28 PROTEIN) \| chr4:14149916-14151104 FORWARD | -3.07 |
|  | 1.1.1.2 | At5g02120 | Symbols: OHP \| OHP (ONE HELIX PROTEIN) \| chr5:419091-419773 FORWARD | -2.843 |
|  | 1.1.1.2 | At5g66570 | Symbols: PSBO-1, OEE1, OEE33, OE33, PSBO1, MSP-1 \| PSBO1 (PS II OXYGEN-EVOLVING COMPLEX 1); oxygen evolving/ poly(U) binding \| chr5:26568653-26570278 FORWARD | -3.155 |
| 3 | 1.1.2.1 | At1g19150 | Symbols: LHCA6, LHCA2*1 \| LHCA6; chlorophyll binding \| chr1:6612749-6613972 FORWARD | -3 |
|  | 1.1.2.1 | At1g61520 | Symbols: LHCA3 \| LHCA3; chlorophyll binding \| chr1:22700010-22701383 FORWARD | -2.82 |
|  | 1.1.2.1 | At3g47470 | Symbols: LHCA4, CAB4 \| LHCA4 (LIGHT-HARVESTING CHLOROPHYLL-PROTEIN COMPLEX I SUBUNIT A4); chlorophyll binding \| chr3:17493372-17495033 REVERSE | -4.002 |
|  | 1.1.2.1 | At3g54890 | Symbols: LHCA1 \| LHCA1; chlorophyll binding \| chr3:20339504-20341103 REVERSE | -2.499 |
|  | 1.1.2.1 | At3g61470 | Symbols: LHCA2 \| LHCA2; chlorophyll binding \| chr3:22745653-22747282 FORWARD | -3.294 |
| 4 | 1.1.2.2 | At1g08380 | Symbols: PSAO \| PSAO (photosystem I subunit O) \| chr1:2640813-2641828 REVERSE | -3.41 |
|  | 1.1.2.2 | At1g30380 | Symbols: PSAK \| PSAK (photosystem I subunit K) \| chr1:10722197-10723247 FORWARD | -2.733 |
|  | 1.1.2.2 | At1g31330 | Symbols: PSAF \| PSAF (photosystem I subunit F) \| chr1:11214824-11216037 REVERSE | -3.766 |
|  | 1.1.2.2 | At1g55670 | Symbols: PSAG \| PSAG (PHOTOSYSTEM I SUBUNIT G) \| chr1:20802670-20803449 REVERSE | -2.88 |
|  | 1.1.2.2 | At2g20260 | Symbols: PSAE-2 \| PSAE-2 (photosystem I subunit E-2); catalytic \| chr2:8736734-8737767 FORWARD | -2.406 |
|  | 1.1.2.2 | At2g46820 | Symbols: PTAC8, TMP14, PSAP, PSI-P \| PSI-P (PHOTOSYSTEM I P SUBUNIT); DNA binding \| chr2:19243589-19245141 FORWARD | -2.91 |
|  | 1.1.2.2 | At4g12800 | Symbols: PSAL \| PSAL (photosystem I subunit L) \| chr4:7521322-7522670 FORWARD | -2.697 |
|  | 1.1.2.2 | At5g64040 | Symbols: PSAN \| PSAN; calmodulin binding \| chr5:25628588-25629615 REVERSE | -3.863 |
| 5 | 1.1.3 | At2g26500 | cytochrome b6f complex subunit (petM), putative \| chr2:11270218-11270874 FORWARD | -2.98 |
| 6 | 1.1.4.7 | At4g09650 | Symbols: ATPD \| ATPD (ATP SYNTHASE DELTA-SUBUNIT GENE); hydrogen ion transporting ATP synthase, rotational mechanism / proton-transporting ATPase, rotational mechanism \| chr4:6100743-6101708 FORWARD | -3.441 |
| 7 | 1.1.5.1 | At1g20340 | Symbols: DRT112, PETE2 \| DRT112; copper ion binding / electron carrier \| chr1:7042429-7043363 REVERSE | -2.467 |
|  | 1.1.5.2 | At1g02180 | ferredoxin-related \| chr1:413522-414645 REVERSE | -4.07 |
|  | 1.1.5.2 | At1g60950 | Symbols: FED A, ATFD2 \| FED A; 2 iron, 2 sulfur cluster binding / electron carrier/ iron-sulfur cluster binding \| chr1:22444520-22445161 FORWARD | -3.233 |
|  | 1.1.5.2 | At3g16250 | Symbols: NDF4 \| NDF4 (NDH-DEPENDENT CYCLIC ELECTRON FLOW 1); electron carrier/ iron-sulfur cluster binding \| chr3:5506931-5508414 REVERSE | -3.731 |
| 8 | 1.1.5.3 | At5g66190 | Symbols: ATLFNR1, FNR1 \| FNR1 (FERREDOXIN-NADP(+)-OXIDOREDUCTASE 1); NADPH dehydrogenase/ electron transporter, transferring electrons within the cyclic electron transport pathway of photosynthesis/ electron transporter, transferring electrons within the noncyclic electron transp \| chr5:26450964-26453199 REVERSE | -3.898 |
| 9 | 1.3.2 | At5g38410 | ribulose bisphosphate carboxylase small chain 3B / RuBisCO small subunit 3B (RBCS-3B) (ATS3B) \| chr5:15377173-15378416 REVERSE | -4.194 |
|  | 1.3.13 | At1g73110 | ribulose bisphosphate carboxylase/oxygenase activase, putative / RuBisCO activase, putative \| chr1:27494121-27496878 REVERSE | -3.506 |
|  | 1.3.13 | At2g39730 | Symbols: RCA \| RCA (RUBISCO ACTIVASE); ADP binding / ATP binding / enzyme regulator/ ribulose-1,5-bisphosphate carboxylase/oxygenase activator \| chr2:16570746-16573548 REVERSE | -4.447 |
| 10 | 1.3.4 | At1g12900 | Symbols: GAPA-2 \| GAPA-2 (GLYCERALDEHYDE 3-PHOSPHATE DEHYDROGENASE A SUBUNIT 2); NAD or NADH binding / binding / catalytic/ glyceraldehyde-3-phosphate dehydrogenase (phosphorylating)/ glyceraldehyde-3-phosphate dehydrogenase \| chr1:4392450-4394400 REVERSE | -3.236 |
|  | 1.3.4 | At1g42970 | Symbols: GAPB \| GAPB (GLYCERALDEHYDE-3-PHOSPHATE DEHYDROGENASE B SUBUNIT); glyceraldehyde-3-phosphate dehydrogenase (NADP+)/ glyceraldehyde-3-phosphate dehydrogenase \| chr1:16127381-16129843 FORWARD | -3.12 |
| 11 | 1.3.6 | At2g21330 | fructose-bisphosphate aldolase, putative \| chr2:9128151-9130240 REVERSE | -4.379 |
|  | 1.3.6 | At4g38970 | fructose-bisphosphate aldolase, putative \| chr4:18163496-18165762 REVERSE | -3.702 |
| 12 | 1.3.7 | At3g54050 | fructose-1,6-bisphosphatase, putative / D-fructose-1,6-bisphosphate 1-phosphohydrolase, putative / FBPase, putative \| chr3:20016905-20018780 FORWARD | -3.451 |
| 13 | 1.3.9 | At3g55800 | Symbols: SBPASE \| SBPASE (sedoheptulose-bisphosphatase); phosphoric ester hydrolase/ sedoheptulose-bisphosphatase \| chr3:20709386-20711640 FORWARD | -4.029 |
| 14 | 1.3.12 | At1g32060 | Symbols: PRK \| PRK (PHOSPHORIBULOKINASE); ATP binding / phosphoribulokinase/ protein binding \| chr1:11532533-11534639 FORWARD | -3.573 |
| 15 | 1.2.2 | At3g14420 | (S)-2-hydroxy-acid oxidase, peroxisomal, putative / glycolate oxidase, putative / short chain alpha-hydroxy acid oxidase, putative \| chr3:4821523-4824178 FORWARD | -3.329 |
| 16 | 1.2.3 | At1g70580 | Symbols: AOAT2, GGT2 \| AOAT2 (ALANINE-2-OXOGLUTARATE AMINOTRANSFERASE 2); L-alanine:2-oxoglutarate aminotransferase/ glycine:2-oxoglutarate aminotransferase \| chr1:26612719-26616069 FORWARD | -2.546 |
|  | 1.2.3 | At2g13360 | Symbols: AGT, AGT1 \| AGT (ALANINE:GLYOXYLATE AMINOTRANSFERASE); alanine-glyoxylate transaminase/ serine-glyoxylate transaminase/ serine-pyruvate transaminase \| chr2:5539240-5541358 REVERSE | -4.514 |
| 17 | 1.2.4.1 | At4g33010 | Symbols: AtGLDP1 \| AtGLDP1 (Arabidopsis thaliana glycine decarboxylase P-protein 1); catalytic/ glycine dehydrogenase (decarboxylating)/ pyridoxal phosphate binding \| chr4:15926670-15931332 REVERSE | -3.064 |
|  | 1.2.4.4 | At1g32470 | glycine cleavage system H protein, mitochondrial, putative \| chr1:11739304-11740361 REVERSE | -2.599 |

**Supplementary Table 3.** Acyl-lipid metabolism transcripts differentially expressed in response to *MGAT1* infiltration.

| **Pathway** | **AGI** | **Protein Family Name** | **Protein Family Abbreviation** | **Isoform/Gene Specific Abbreviation** | **LogFC** |
| --- | --- | --- | --- | --- | --- |
| Cutin Synthesis & Transport 1 | AT3G04290 | Polyester Synthase | PS | LTL1=>LTL1/CD1 | -6.59694 |
| Cutin Synthesis & Transport 1 | AT5G33370 | Polyester Synthase | PS |  | -4.07039 |
| Cutin Synthesis & Transport 1 | AT5G25390 | SHN Transcription Factors | Regulatory protein | SHN3 | -5.39689 |
| Cutin Synthesis & Transport 1 | AT2G26910 | ABC Transporter | ABC | ABCG32/PEC1 | -3.18057 |
| Cutin Synthesis & Transport 1 | AT5G23940 | DCR/PEL3 Acyltransferase | Unknown function | PEL3/DCR | -3.69437 |
| Cutin Synthesis & Transport 1 | AT2G45970 | Fatty Acyl omega-Hydroxylase | FAH | CYP86A8/LCR | -5.20163 |
| Cutin Synthesis & Transport 1 | AT4G00360 | Fatty Acyl omega-Hydroxylase | FAH | CYP86A2 | -4.65535 |
| Cutin Synthesis & Transport 1 | AT1G49430 | Long-Chain Acyl-CoA Synthetase | LACS | LACS2 | -4.02203 |
| Cutin Synthesis & Transport 1 | AT1G72970 | Omega-Hydroxy Fatty Acyl Dehydrogenase | HFADH | HTD | -3.23266 |
| Cutin Synthesis & Transport 2 | AT1G17840 | ABC Transporter | ABC | WBC11 / ABCG11 / DSO/COF1 | -3.31667 |
| Eukaryotic Galactolipid & Sulfolipid Synthesis | AT2G26870 | Phospholipase C (Non specific) | nsPLC | NPC2 (Non specific) | -3.48543 |
| Eukaryotic Galactolipid & Sulfolipid Synthesis | AT3G48610 | Phospholipase C (Non specific) | nsPLC | NPC6 (Non specific) | -3.60407 |
| Eukaryotic Phospholipid Synthesis & Editing | AT2G29980 | Linoleate Desaturase | FAD3 | FAD3 | -2.75987 |
| Eukaryotic Phospholipid Synthesis & Editing | AT2G41540 | NAD-dependent Glycerol-3-Phosphate Dehydrogenase | GPDH | GPDHc1 | 2.514573 |
| Eukaryotic Phospholipid Synthesis & Editing | AT2G41540 | NAD-dependent Glycerol-3-Phosphate Dehydrogenase | GPDH | GPDHc1 | -3.17206 |
| Eukaryotic Phospholipid Synthesis & Editing | AT1G48600 | Phosphoethanolamine N-Methyltransferase | PEAMT | CPUORF31 | -2.74108 |
| Eukaryotic Phospholipid Synthesis & Editing | AT1G73600 | Phosphoethanolamine N-Methyltransferase | PEAMT |  | -3.88753 |
| Fatty Acid Elongation & Wax Biosynthesis | AT3G21090 | ABC Transporter | ABC | WBC15/22 / ABCG15 | -3.35418 |
| Fatty Acid Elongation & Wax Biosynthesis | AT3G52310 | ABC Transporter | ABC | WBC28 / ABCG27 | -2.67205 |
| Fatty Acid Elongation & Wax Biosynthesis | AT5G06530 | ABC Transporter | ABC | WBC23 / ABCG22 | -4.33832 |
| Fatty Acid Elongation & Wax Biosynthesis | AT1G17840 | ABC Transporter | ABC | WBC11 / ABCG11 / DSO/COF1 | -3.31667 |
| Fatty Acid Elongation & Wax Biosynthesis | AT3G49190 | Bifunctional Wax Ester Synthase / Diacylglycerol Acyltransferase | WSD |  | -3.9855 |
| Fatty Acid Elongation & Wax Biosynthesis | AT3G23840 | CER2-like Protein | CER2-like |  | -3.43589 |
| Fatty Acid Elongation & Wax Biosynthesis | AT5G57800 | CER3 Protein | CER3 | CER3/WAX2/YRE/FLP1 | -2.51728 |
| Fatty Acid Elongation & Wax Biosynthesis | AT5G59770 | Hydroxyacyl-CoA Dehydratase | HACD |  | -2.85561 |
| Fatty Acid Elongation & Wax Biosynthesis | AT1G68530 | Ketoacyl-CoA Synthase | KCS | KCS6 | -3.04626 |
| Fatty Acid Elongation & Wax Biosynthesis | AT2G38540 | Lipid Transfer Protein | LTP | (LTP type 1) | -2.90903 |
| Fatty Acid Elongation & Wax Biosynthesis | AT3G08770 | Lipid Transfer Protein | LTP | (LTP type 1) | -3.21266 |
| Fatty Acid Elongation & Wax Biosynthesis | AT5G59320 | Lipid Transfer Protein | LTP | (LTP type 1) | -4.77961 |
| Fatty Acid Elongation & Wax Biosynthesis | AT3G18280 | Lipid Transfer Protein | LTP | (LTP type 2) | -2.45066 |
| Fatty Acid Elongation & Wax Biosynthesis | AT1G27950 | Lipid Transfer Protein | LTP | LTPG1 (LTP type 5) | -2.99474 |
| Fatty Acid Elongation & Wax Biosynthesis | AT2G44300 | Lipid Transfer Protein | LTP | (LTP type 5) | -4.47335 |
| Fatty Acid Elongation & Wax Biosynthesis | AT3G43720 | Lipid Transfer Protein | LTP | (LTP type 5) | -2.69471 |
| Fatty Acid Elongation & Wax Biosynthesis | AT1G49430 | Long-Chain Acyl-CoA Synthetase | LACS | LACS2 | -4.02203 |
| Fatty Acid Elongation & Wax Biosynthesis | AT2G23180 | Midchain Alkane Hydroxylase | CYP450, 96A | CYP96A1 | -2.75048 |
| Fatty Acid Elongation & Wax Biosynthesis | AT5G25390 | SHN Transcription Factors | Regulatory protein | SHN3 | -5.39689 |
| Fatty Acid Elongation and Wax Biosynthesis | AT3G54010 | Immunophilin-like FK506 binding protein | Regulatory protein | PAS1/DEI1 | -2.45337 |
| Fatty Acid Elongation and Wax Biosynthesis | AT3G61150 | Homeodomain-Leucine Zipper IV | Regulatory protein | HDG1 | -3.7137 |
| Fatty Acid Elongation, Desaturation  & Export From Plastid | AT1G74960 | Ketoacyl-ACP Synthase II | KAS II | KASII | -2.56618 |
| Fatty Acid Synthesis | AT5G46290 | Ketoacyl-ACP Synthase I | KASI | KASI | -2.34303 |
| Fatty Acid Synthesis; Prokaryotic Galactolipid,  Sulfolipid, & Phospholipid Synthesis 1 | AT1G74960 | Ketoacyl-ACP Synthase II | KASII | KASII | -2.56618 |
| Mitochondrial Fatty Acid & Lipoic Acid Synthesis | AT3G16170 | Malonyl-CoA Synthase | MCS | MCS/AAE13 | -2.66448 |
| Mitochondrial Phospholipid Synthesis | AT1G63430 | CDP-DAG Synthase | CDP-DAGS | CDS1; CDP-DAGS | -2.37141 |
| Oxylipin Metabolism 1 | AT2G39220 | Acyl-Hydrolase (Patatin-like) | PAH |  | -2.47454 |
| Oxylipin Metabolism 1 | AT3G54950 | Acyl-Hydrolase (Patatin-like) | PAH |  | -3.21604 |
| Oxylipin Metabolism 1 | AT4G15440 | Hydroperoxide Lyase | HPL |  | -3.65076 |
| Oxylipin Metabolism 1 | AT1G19640 | Jasmonic Acid Carboxyl Methyltransferase | JCMT |  | -3.88753 |
| Oxylipin Metabolism 1; Oxylipin Metabolism 2 | AT2G42690 | Acylhydrolase (DAD1-like) | AH |  | -3.63227 |
| Oxylipin Metabolism 1; Oxylipin Metabolism 2 | AT4G18550 | Acylhydrolase (DAD1-like) | AH |  | -2.35529 |
| Oxylipin Metabolism 1; Oxylipin Metabolism 2 | AT3G14075 | Lipid Acylhydrolase-like | LAH |  | 2.714246 |
| Oxylipin Metabolism 1; Oxylipin Metabolism 2 | AT5G37710 | Lipid Acylhydrolase-like | LAH |  | 3.865668 |
| Oxylipin Metabolism 1; Oxylipin Metabolism 2 | AT1G55020 | Lipoxygenase | LOX |  | -3.26303 |
| Oxylipin Metabolism 1; Oxylipin Metabolism 2 | AT3G22400 | Lipoxygenase | LOX |  | 2.625709 |
| Oxylipin Metabolism 1; Oxylipin Metabolism 2 | AT5G42930 | Lipid Acylhydrolase-like | LAH |  | -2.4975 |
| Pathway, function or subcellular  location uncertain | AT4G04320 | Malonyl-CoA Decarboxylase | MCD |  | 2.906891 |
| Pathway, function or subcellular  location uncertain | AT1G59820 | Translocase | TL |  | 2.415904 |
| Pathway, function or subcellular  location uncertain | AT3G27870 | Translocase | TL |  | -3.37851 |
| Phospholipid Signaling | AT2G20900 | Diacylglycerol Kinase | DAGK |  | 2.807355 |
| Phospholipid Signaling | AT4G38690 | Glycosylphosphatidylinositol-specific Phospholipase C | GPI-PLC |  | -3.03562 |
| Phospholipid Signaling | AT3G10840 | Lysophospholipase | LPLA |  | -3.20163 |
| Phospholipid Signaling | AT3G15650 | Lysophospholipase | LPLA |  | -2.60407 |
| Phospholipid Signaling | AT5G19290 | Lysophospholipase | LPLA |  | -2.5717 |
| Phospholipid Signaling | AT5G20060 | Lysophospholipase | LPLA |  | -3.43296 |
| Phospholipid Signaling | AT1G64470 | Phosphatidylinositol-4-Kinase gamma | PI4K gamma |  | -2.54314 |
| Phospholipid Signaling | AT2G46500 | Phosphatidylinositol-4-Kinase gamma | PI4K gamma |  | -3.30256 |
| Phospholipid Signaling | AT5G24240 | Phosphatidylinositol-4-Kinase gamma | PI4K gamma |  | -3.37851 |
| Phospholipid Signaling | AT1G60890 | Phosphatidylinositol-Phosphate Kinase type IB | PIPK-IB |  | -3.07192 |
| Phospholipid Signaling | AT3G19240 | Phosphoinositide 3-Phosphatase | PI3P |  | 3.279173 |
| Phospholipid Signaling | AT2G40116 | Phosphoinositide-specific Phospholipase C | PIPLC |  | -3.31034 |
| Phospholipid Signaling | AT3G08510 | Phosphoinositide-specific Phospholipase C | PIPLC |  | -3.04731 |
| Phospholipid Signaling | AT3G55940 | Phosphoinositide-specific Phospholipase C | PIPLC |  | -3.77493 |
| Phospholipid Signaling | AT2G42010 | Phospholipase D beta | PLD beta |  | 3.242494 |
| Phospholipid Signaling | AT4G35790 | Phospholipase D delta | PLD delta |  | 2.78136 |
| Prokaryotic Galactolipid,  Sulfolipid, & Phospholipid Synthesis 2 | AT3G15850 | FAD5-like Desaturase | FAD5 like (ADS) | FAD5/ADS3 | -2.72247 |
| Sphingolipid Biosynthesis 1 | AT2G34770 | Fatty Acid 2-hydroxylase | FA2H | FAH1 | -3.74846 |
| Sphingolipid Biosynthesis 1 | AT2G46210 | Sphingobase-D8 Desaturase | SLD | SLD2 | -5.16992 |
| Suberin Synthesis & Transport 1 | AT1G68530 | Ketoacyl-CoA Synthase | KCS | KCS6/CER6/CUT1 | -3.04626 |
| Suberin Synthesis & Transport 1 | AT1G49430 | Long-Chain Acyl-CoA Synthetase | LACS | LACS2 | -4.02203 |
| Suberin Synthesis & Transport 1 | AT1G12570 | omega-Hydroxy Fatty Acyl Dehydrogenase | HFADH |  | -5.80735 |
| Suberin Synthesis & Transport 1 | AT1G72970 | omega-Hydroxy Fatty Acyl Dehydrogenase | HFADH | HTD | -3.23266 |
| Suberin Synthesis & Transport 1 | AT5G51950 | omega-Hydroxy Fatty Acyl Dehydrogenase | HFADH |  | -3.48543 |
| Suberin Synthesis & Transport 1 | AT5G25390 | SHN Transcription Factors | Regulatory protein | SHN3 | -5.39689 |
| Suberin Synthesis & Transport 1 | AT1G01610 | Glycerol-3-Phosphate Acyltransferase | GPAT | sn-2-GPAT4 | -4.58601 |
| Suberin Synthesis & Transport 1 | AT1G01610 | Glycerol-3-Phosphate Acyltransferase | GPAT | sn-2-GPAT4 | -4.58601 |
| Suberin Synthesis & Transport 3 | AT1G17840 | ABC transporter | ABC | WBC11 / ABCG11 / DSO/COF1 | -3.31667 |
| Suberin Synthesis & Transport 3 | AT1G27950 | Lipid Transfer Protein | LTP | LTPG1 (LTP type 5) | -2.99474 |
| Triacylglycerol & Fatty Acid Degradation | AT1G18360 | Monoacylglycerol Lipase (MAGL) | MAGL |  | -2.76553 |
| Triacylglycerol & Fatty Acid Degradation | AT5G19290 | Monoacylglycerol Lipase (MAGL) | MAGL |  | -2.5717 |
| Triacylglycerol & Fatty Acid Degradation | AT5G42930 | Triacylglycerol Lipase (TAGL) | TAGL |  | -2.4975 |
| Triacylglycerol Biosynthesis | AT2G19450 | Acyl-CoA : Diacylglycerol Acyltransferase | DGAT | DGAT1 | 2.933837 |
| Triacylglycerol Biosynthesis | AT2G29980 | Linoleate Desaturase | FAD3 | FAD3 | -2.75987 |
| Triacylglycerol Biosynthesis | AT2G41540 | NAD-dependent Glycerol-3-Phosphate Dehydrogenase | GPDH | GPDHc1 | 2.514573 |
| Triacylglycerol Biosynthesis | AT2G41540 | NAD-dependent Glycerol-3-Phosphate Dehydrogenase | GPDH | GPDHc1 | -3.17206 |
| Triacylglycerol Biosynthesis | AT3G15820 | Phosphatidylcholine:diacylglycerol cholinephosphotransferase | PDCT | PDCT/ROD1 | 4.321928 |

**Supplememtary Table 4.** Primer sequences and description of genes used for qRT-PCR analysis.

| Target sequence ID | Annotation | Sequence 5'-3' |
| --- | --- | --- |
| CL1305.Contig1_Benth | DGAT1 (diacylglycerol acyltranferase) | TCATGCAGGCCTACTCAATC |
|  |  | AAGACAACACAGCAGAAGTGG |
| unigene66433 | CML37 ,(calmodulin like 37) | CACCGAGTCGACTCAGCATCCTCT |
|  |  | GCGATGGATTGTTGGGTTTGGAG |
| unigene7917 | HSP101(heat shock protein 101) | GCCTCGTTAGTCTTGTGGGT |
|  |  | TACACAAAGCGACTCCAAGC |
| CL703.Contig1_Benth | NCED4 (nine-cis-epoxycarotenoid dioxygenase 4) | TGCGTCTGTTGAGAAAGTGA |
|  |  | CTATCCTTGGCATTGGGTCT |
| *N. benthmiana* *GAPDH* | GAPDH | CACTACCAACTGCCTTGCAC |
|  |  | ATGAAGCAGCTCTTCCACCT |
| N. benthmiana *Actin* | Actin | TCCCATTGTGCAATTCATTC |
|  |  | AGCCTTGACCATTCCTGTTC |

## 2.2. Supplementary Figures
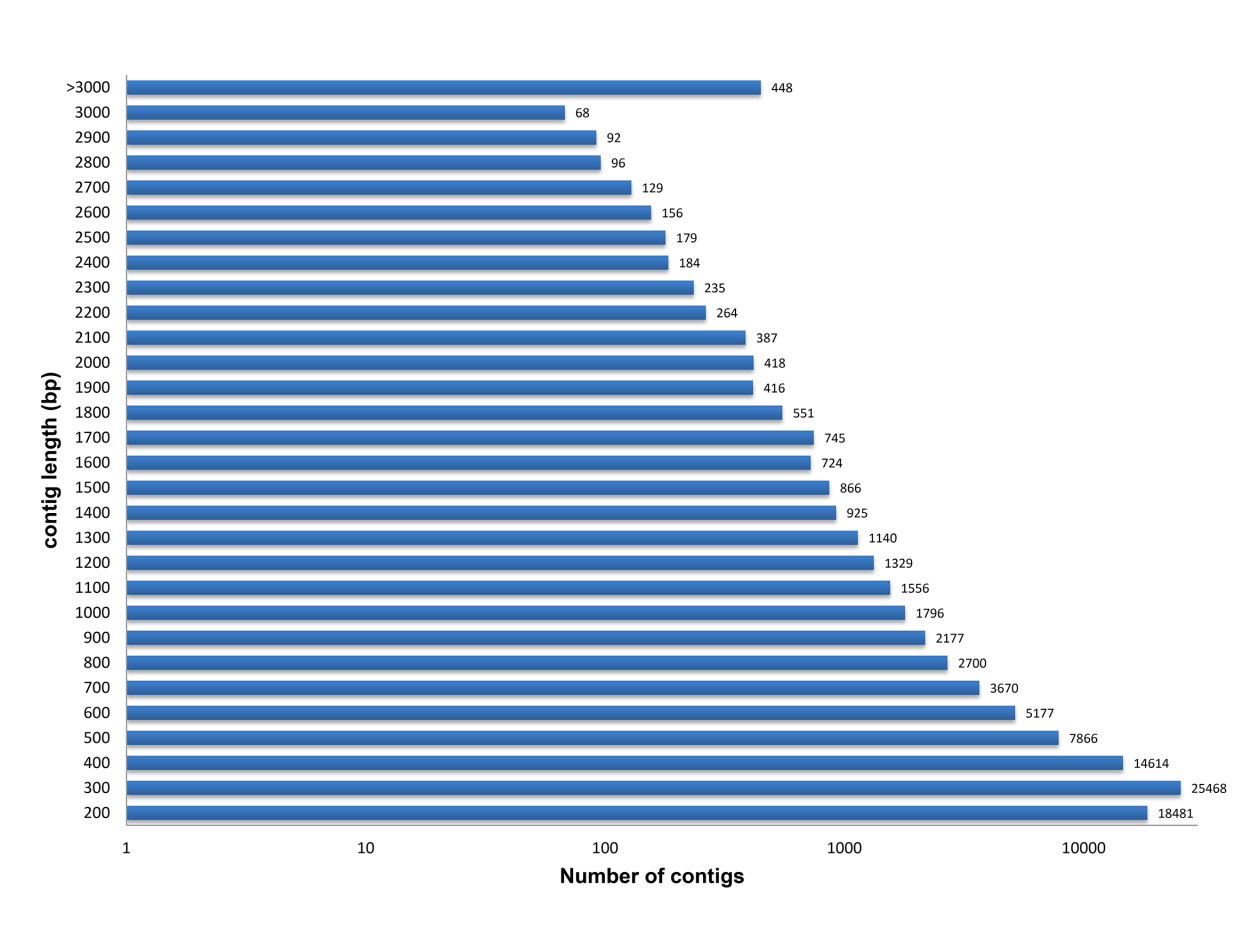


**Supplementary Figure 1**. Length distribution of *N. benthamiana* contigs generated from *de novo* assembly.


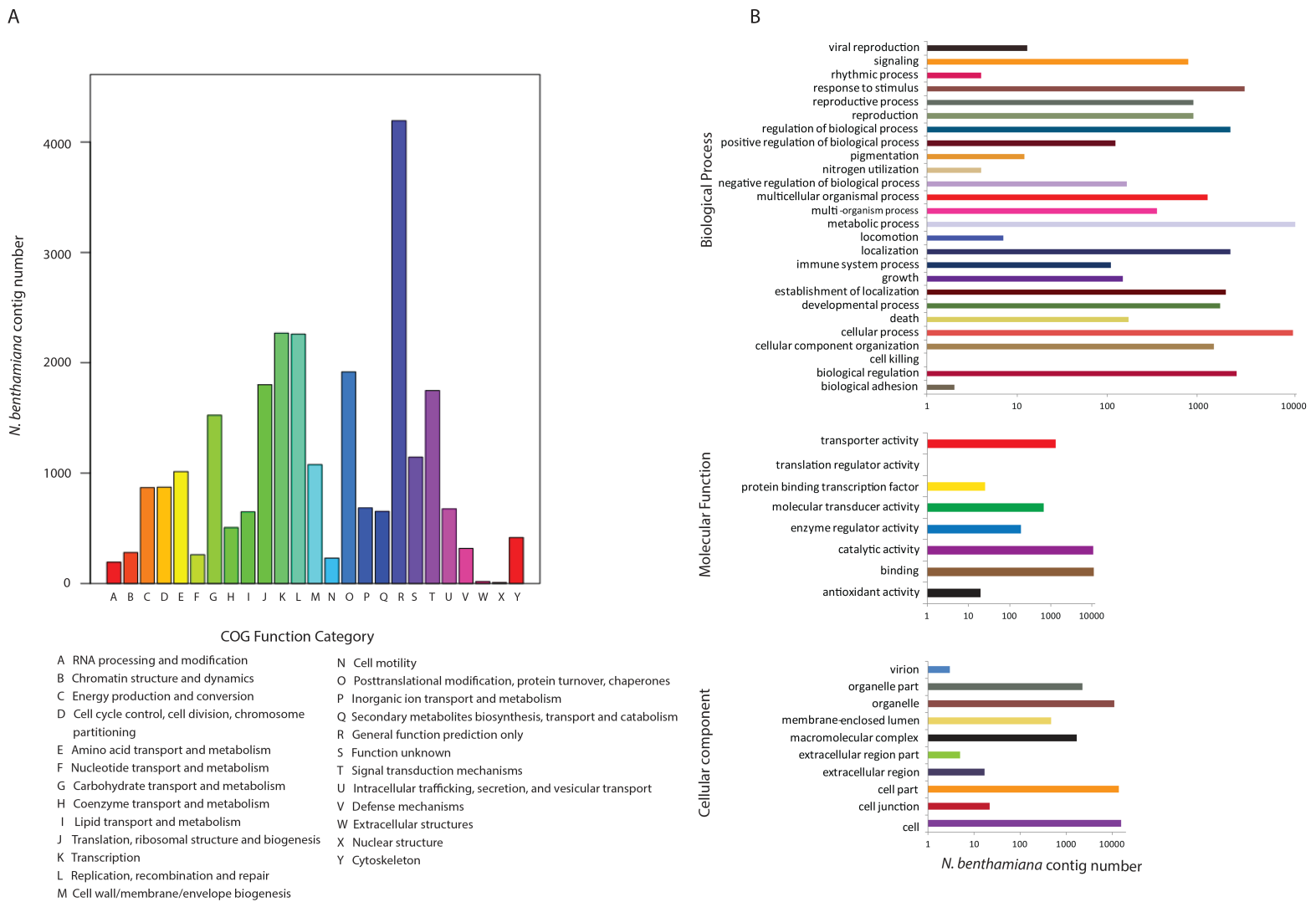


**Supplementary Figure 2**. Functional categorization of *N. benthamiana* contigs. A) Clusters of orthologous genes (COG) functional classification. B) Gene Ontology (GO) term annotation showing distribution of contigs into biological process, molecular function and cellular component.


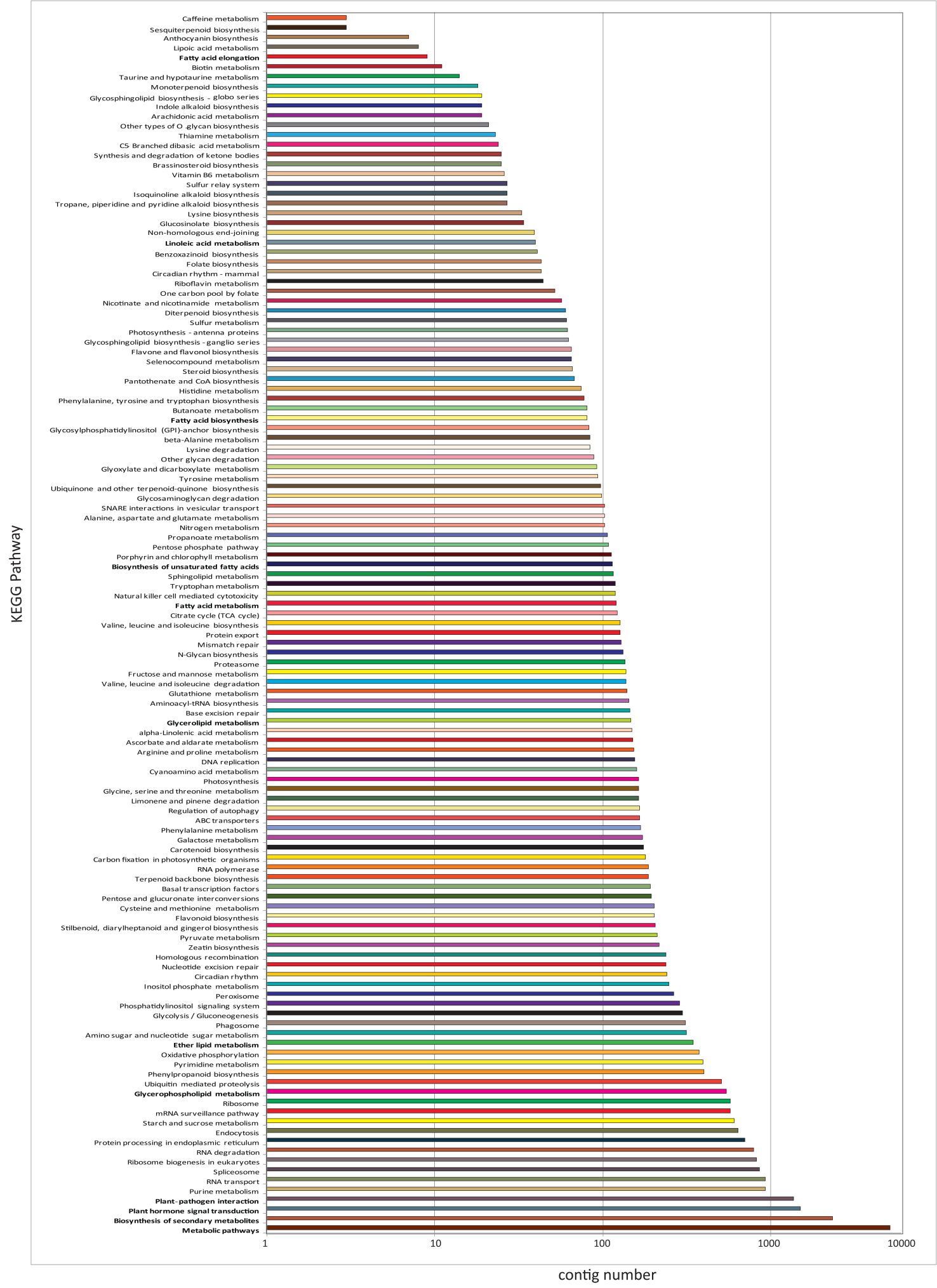


**Supplementary Figure 3.** Metabolic pathway annotations by Kyoto Encyclopedia of Genes and Genomes database (KEGG). Enriched pathways and number of *N. benthamiana* contigs in each pathway are depicted.

**
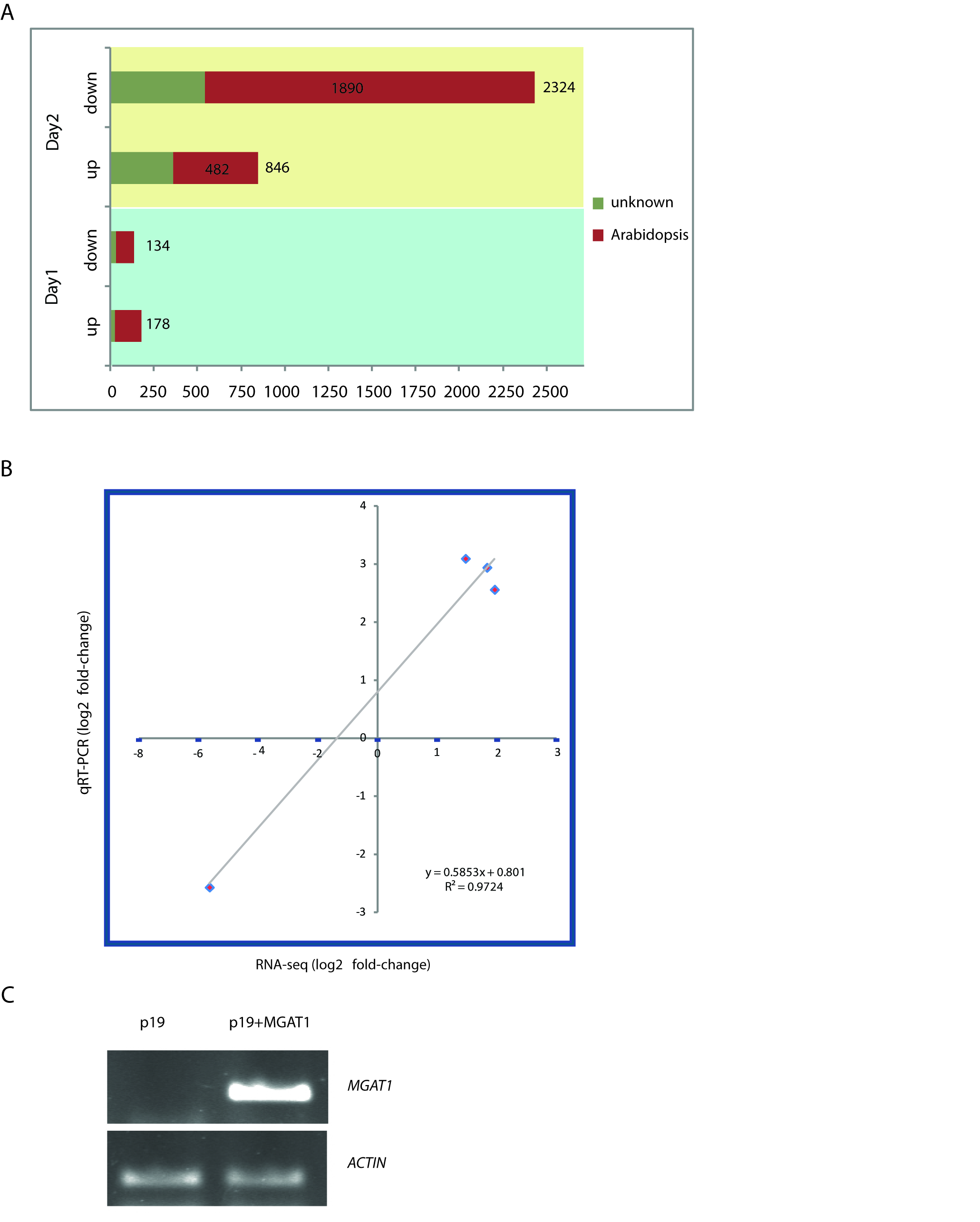
**

**Supplementary Figure 4.** Genes differentially expressed by MGAT1 infiltration. A) Number of transcripts up- or down-regulated at 1 dpi and 2 dpi. Total number of transcripts changed is indicated by the bar. Green portion indicates transcript number with no *A. thaliana* homologues. Red portion indicates the number of transcripts with *A. thaliana* homologues. B) qRT-PCR validation of RNA-seq. Log2 fold-change values of four genes obtained by RNA-seq were plotted against their qRT-PCR Log2 fold-change values. Genes used were CL1305.Contig1_Benth, unigene66433, unigene7917, CL703.Contig1_Benth. The *N. benthamiana* *GAPDH* and *ACTIN* genes were used as internal controls. C) RT-PCR showing transient accumulation of *MGAT1* transcripts in the infiltrated leaves.


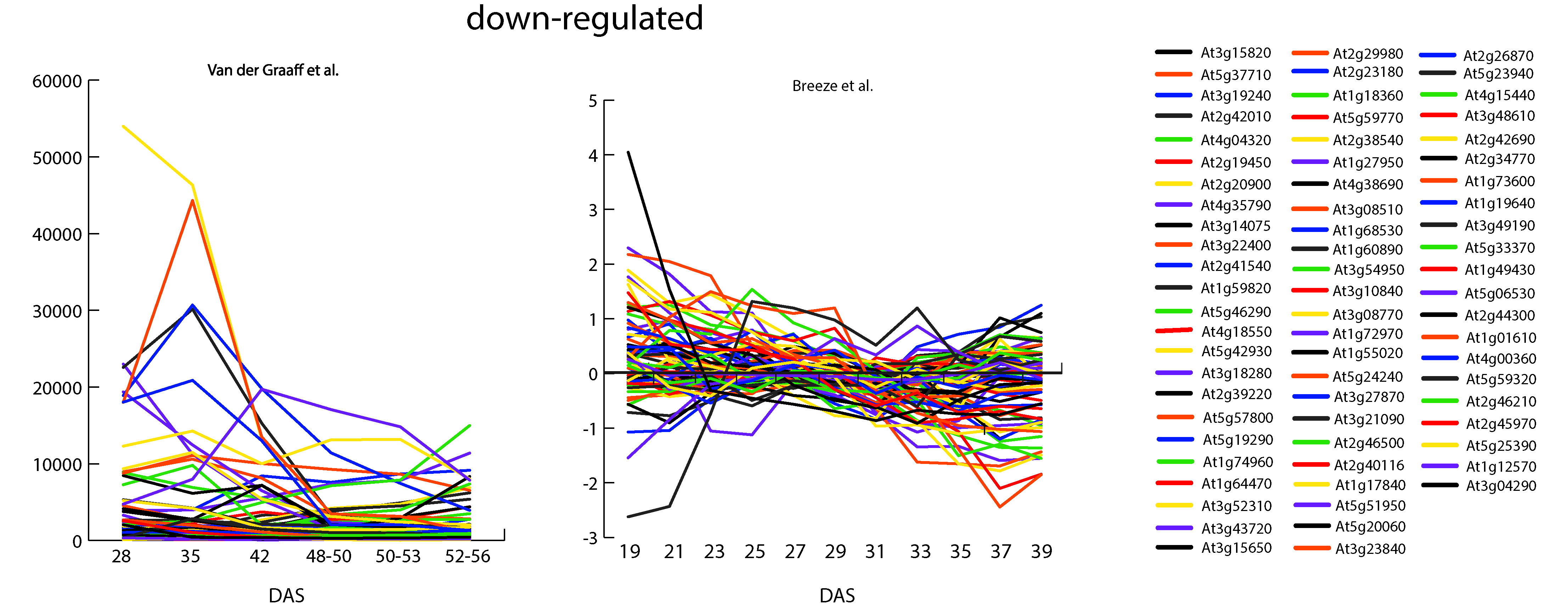


**Supplementary Figure 5.** Expression profiles of lipid related genes down-regulated by *MGAT1*. Majority of down-regulated genes were also down-regulated in both senescence studies (Breeze et al. 2011 and Vander der Graff et al. 2006).


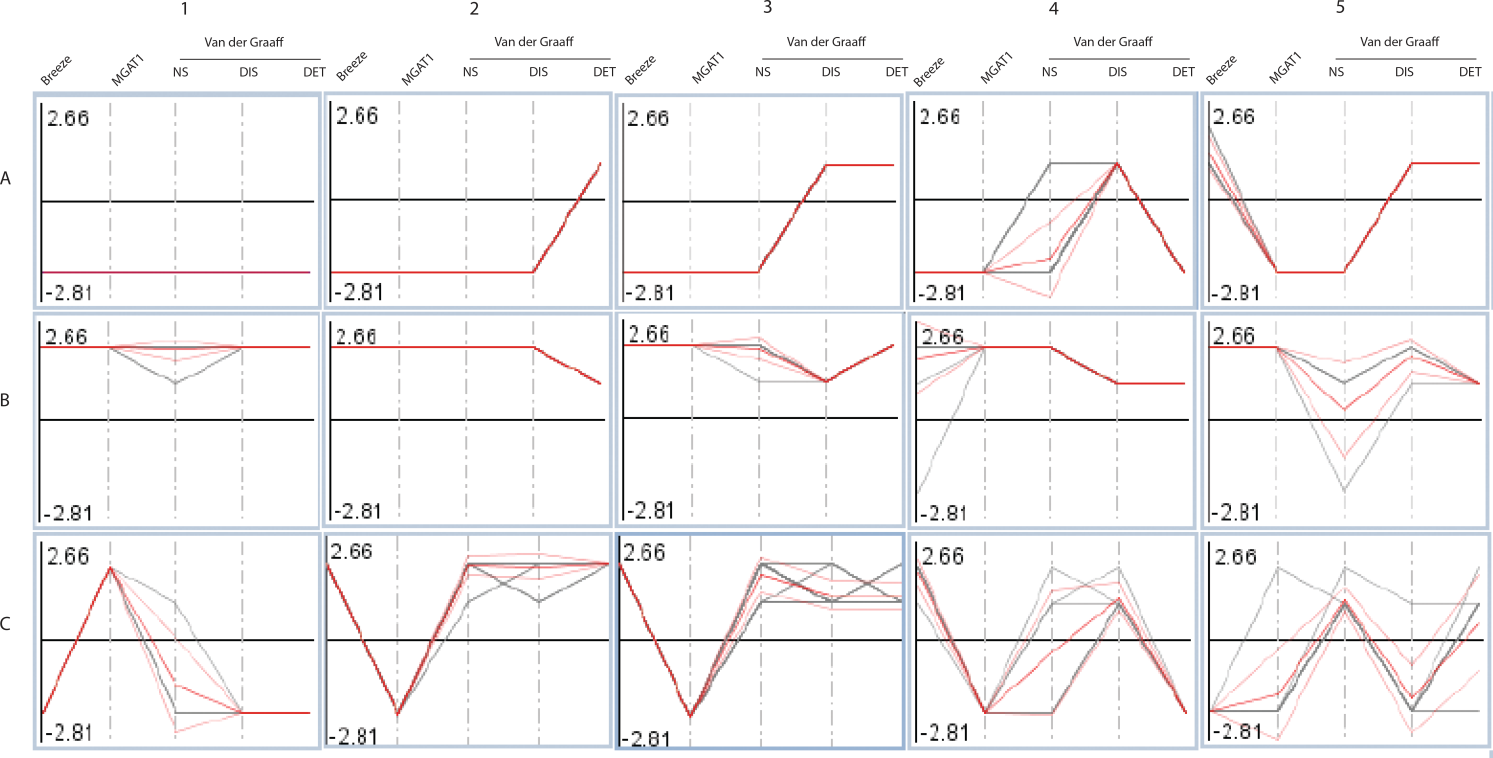


**Supplementary Figure 6.** Comparative analysis of *MGAT1* and senescence responsive transcriptome. MapMan cluster analysis of transcripts common to *MAGT1*, Breeze *et al* and Van der Graaff *et al.* Up-regulated genes were given a value of 2, down-regulated genes a value of -2 and a value of 1 is given when no change in expression is observed. Eucledian distance metric was employed to generate 15 clusters. NS: developmental leaf senescence; DIS: darkening-induced senescence in intact leaves; DET: senescence in dark-incubated detached leaves.
